# Canagliflozin Ameliorates NLRP3 Inflammasome-Mediated Inflammation Through Inhibiting NF-κB Signaling and Upregulating Bif-1

**DOI:** 10.3389/fphar.2022.820541

**Published:** 2022-03-28

**Authors:** Yaoyun Niu, Yuehui Zhang, Wanqiu Zhang, Jinghua Lu, Yang Chen, Wenhui Hao, Jin Zhou, Lijun Wang, Weidong Xie

**Affiliations:** ^1^ State Key Laboratory of Chemical Oncogenomics, Shenzhen International Graduate School, Tsinghua University, Shenzhen, China; ^2^ Shenzhen Key Lab of Health Science and Technology, Institute of Biopharmaceutical and Health Engineering, Shenzhen International Graduate School, Tsinghua University, Shenzhen, China; ^3^ Department of Critical Care Medicine, The People’s Hospital of Baoan, Shenzhen, China; ^4^ Department of Critical Care Medicine, Second Affiliated Hospital of Shenzhen University, Shenzhen, China; ^5^ The Second School of Clinical Medicine, Southern Medical University, Shenzhen, China; ^6^ Institute for Ocean Engineering, Shenzhen International Graduate School, Tsinghua University, Shenzhen, China

**Keywords:** canagliflozin, NLPR3, inflammasome, NF-κB, Bif-1, pneumonia

## Abstract

NOD-, LRR-, and pyrin domain-containing protein 3 (NLRP3) inflammasome is an important component of the innate immune system that mediates the secretion of the pro-inflammatory cytokines interleukin-1β (IL-1β) and IL-18. However, current studies have shown that the abnormal activation of the NLRP3 inflammasome is associated with inflammatory diseases such as atherosclerosis, diabetes, and pneumonia. In this study, we found that canagliflozin (CAN) transcriptionally inhibited NLRP3 inflammasome-related proteins by inhibiting the transduction of the nuclear factor κB signal. Autophagy is largely involved in the post-translational modifications of the NLRP3 inflammasome and is an important regulator of NLRP3 inflammasome assembly and activation. Bax-interacting factor 1 (Bif-1) plays an important role in autophagosome formation during early-stage autophagy. Our results are the first to indicate that CAN, a hypoglycemic drug, can inhibit the activation of NLRP3 inflammasome and inflammation by upregulating Bif-1 and autophagy in a non-hypoglycemic manner. This study provides new information regarding the treatment of patients with pneumonia, particularly those with concurrent diabetes.

## Introduction

Interleukin-1β (IL-1β) is secreted by a variety of cells, the most important of which are the macrophages. It is a pro-inflammatory cytokine that plays an important role in the host defense response to infections (Dinarello, 1996). It is produced as an inactive 31 kDa precursor, termed pro-IL-1β, in response to pathogen-associated molecular patterns (PAMPs). Pattern recognition receptors on the surface of the macrophages activate pathways that control IL-1β transcription after recognizing PAMPs (Lopez-Castejon and Brough, 2011). Pro-IL-1β is processed by a protein complex called the inflammasome and is cleaved into an active 17 kDa form of IL-1β. NOD-, LRR-, and pyrin domain-containing protein 3 (NLRP3) inflammasome is the most widely studied inflammasome. The NLRP3 inflammasome consists of a sensor NLRP3, an adaptor ASC, and an effector caspase-1. Activation of the NLRP3 inflammasome depends on two signals. Signal 1, or the priming signal prompted by the activation of PAMPs, leads to nuclear factor κB (NF-κB)-dependent transcriptional upregulation of the NLPR3 inflammasome components (Cornut et al., 2020). Signal 2, or the activation signal prompted by PAMPs or damage-associated molecular patterns, such as adenosine triphosphate (ATP), activate multiple upstream signaling events. Once activated, NLRP3 recruits ASC and nucleates the helical ASC filament. The assembled ASC recruits caspase-1 and enables proximity-induced caspase-1 self-cleavage. The activated form of caspase-1 cleaves pro-IL-1β and pro-interleukin-18 into their active forms (Swanson et al., 2019).

Autophagy is an important mechanism by which cells degrade damaged proteins and organelles (Ma et al., 2013). It is also an important pathway for immune cells to degrade foreign pathogens. Lipopolysaccharide (LPS) treatment induces autophagy in macrophages and enhances the co-localization of intracellular bacteria and autophagosomes (Xu et al., 2007). In addition, increasing evidence has shown that autophagy is largely involved in the post-translational modifications of the NLRP3 inflammasome and is an important regulator of NLRP3 inflammasome assembly and activation (Lopez-Castejon, 2020). NLRP3 (Spalinger et al., 2017) and ASC (Shi et al., 2012; Spalinger et al., 2017) were found in the autophagosomes of activated macrophages, indicating that autophagy may be a negative regulator of the NLRP3 inflammasome.

Canagliflozin (CAN) is a small-molecule hypoglycemic drug that inhibits renal glucose reabsorption by inhibiting the type 2 sodium-glucose cotransporter (SGLT-2), thereby, reducing blood glucose concentration (Nomura et al., 2010). In addition to the hypoglycemic effect, CAN is involved in a few activities that are independent of SGLT-2. Several studies and clinical trials have shown that CAN reduces the risk of cardiovascular events (Neal et al., 2017; Kosiborod et al., 2018; Mahaffey et al., 2018; Sun et al., 2021). We previously found that CAN effectively alleviated the pathological changes in lung inflammation in LPS-treated NIH mice (Xu et al., 2018; Niu et al., 2021) and reduced the level of IL-1β in lung tissue and plasma (Niu et al., 2021). However, the specific mechanism is still unclear.

We found that CAN can exert anti-inflammatory effects by promoting autophagy. Treatment with the autophagy inhibitor 3-MA, which targets PI3KC3, reversed the anti-inflammatory effect of CAN (Xu et al., 2018). This suggests that CAN may affect regions upstream of PI3KC3. Bif-1, also known as SH3GLB1 or endophilin B1, plays an important role in the formation of autophagosomes during early autophagy (Takahashi et al., 2007). It interacts with Beclin-1 through ultraviolet radiation resistance-associated gene and positively regulates PI3KC3 (Takahashi et al., 2007). In this study, we unexpectedly found that the knockdown of Bif-1 reversed the effect of CAN, suggesting that CAN can inhibit NLRP3 inflammasome activation depending on Bif-1-related autophagy. Therefore, in this study, we investigated the mechanisms underlying the anti-inflammatory effects of CAN on inflammasomes and Bif-1-related autophagy.

## Materials and Methods

### Mice

Four-week-old NIH Swiss male mice (derived from National Institutes of Health) were purchased from Guangdong Medical Laboratory Animal Center and housed under controlled conditions (constant temperature: 22 ± 2°C; constant humidity: 60 ± 5%; 12-h dark/light cycle). The study was conducted in strict accordance with the National Institutes of Health’s Guide for the Care and Use of Laboratory Animals, and the protocol was approved by the Bioethics Committee of the Shenzhen International Graduate School, Tsinghua University, China. The mice were divided into the following groups: normal control, untreated LPS control (10 mg/kg, Sigma-Aldrich, United States), and CAN (20 mg/kg, Biochempartner, Shanghai, China).

CAN was dissolved in 0.5% (w/v) sodium carboxymethylcellulose (CMC, Sangon Biotech, Shanghai, China) and administered intragastrically for 3 consecutive days. The normal and model groups were administered the same amount of the vehicle. After treatment, the mice were intraperitoneally injected with LPS (10 mg/kg). After 4 h of LPS induction, mice were anesthetized by intraperitoneal injection of 10% (w/v) urethane solution (10 ml/kg; dissolved in normal saline; Sangon Biotech). Blood samples were collected and stored at −80°C for further research. The mice were sacrificed by cervical dislocation, and the lung tissues were removed. Some of the lung tissues were homogenized in 1 ml of ice-cold lysis buffer [50 mM Tris (Sangon Biotech, China), 150 mM NaCl (Sangon Biotech), 0.1% (w/v) sodium dodecyl sulfate (Sangon Biotech), 1% Triton X-100 (Sangon Biotech), and 1 tablet/50 ml Protease Inhibitor Cocktail (Roche, Switzerland), pH 8.0]. Total ribonucleic acid (RNA) from lung tissue was extracted using RNAiso Plus (TaKaRa Biotechnology, China) following the manufacturer’s instructions. The remaining lung tissues were stored at −80°C.

### Cell Culture and Treatment

J774A.1 (Procell CL-0370) was kindly provided by Procell Life Science & Technology Co., Ltd., Wuhan, China. The cells were grown in RPMI 1640 medium (Gibco, Thermo Fisher Scientific, United States) supplemented with 10% fetal bovine serum (Premium, Pan Biotech, Germany) and 1% penicillin-streptomycin antibiotic (Gibco, Thermo Fisher Scientific).

For inducing NLRP3-inflammasome activation, cells were primed with LPS (500 ng/ml, Sigma-Aldrich, United States) for 6 h in the presence or absence of CAN (10 μM, Biochempartner). Inhibitors used in the study (if any) were also added simultaneously with LPS and CAN. Then, stimulated with ATP (5 mM, Sigma-Aldrich, United States) for 30 min.

The supernatant was collected for either the enzyme-linked immunosorbent assay (ELISA) or western blotting experiments after being concentrated in ultrafiltration centrifuge tubes (3 kDa, Merck, Germany). Cells were washed twice with ice-cold phosphate-buffered saline (Sangon Biotech), and 1 ml of RNA isolation reagent (TRIzol, Invitrogen, Thermo Fisher Scientific) or 80 μl of lysis buffer [50 mM Tris (Sangon Biotech), 150 mM NaCl (Sangon Biotech), 0.1% (w/v) sodium dodecyl sulfate (Sangon Biotech), 1% Triton X-100 (Sangon Biotech), and 1 tablet/50 ml Protease Inhibitor Cocktail (Roche), pH 8.0] was added for cell RNA or protein extraction, respectively.

### siRNA Transfection

J774A.1 cells were grown in 6-well plates to 50% confluence. For each well, 250 μl transfection reagent [5 μl Lipofectamine 3000 Reagent (Thermo Fisher Scientific) and 245 μl Opti-MEM (Gibco, Thermo Fisher Scientific)] and 250 μl siRNA solution (100 nM, si-Sh3glb1, RIBOBIO, China; diluted with Opti-MEM) were prepared. These solutions were mixed by pipetting and incubated for 20 min at room temperature. The cells were washed twice with PBS, and 1.5 ml Opti-MEM and the mixed solution were added to each well. Twenty-four hours after transfection, cells were primed with LPS (500 ng/ml) for 6 h in the presence or absence of CAN (10 μM) and then stimulated with 5 mM ATP for 30 min. Whole cell lysis and supernatants were collected for further assays.

### Western Blotting

Protein samples were separated using sodium dodecyl sulfate-polyacrylamide gel electrophoresis (Epizyme Biotech, China) and transferred to nitrocellulose membranes (Bio Trace, New Zealand). The membranes were blocked with freshly prepared 5% (w/v) nonfat dry milk (Anchor, New Zealand) dissolved in tris-buffered saline with 0.1% tween 20 (TBST) for 2 h and incubated overnight at 4°C with primary antibodies dissolved in 1.5% bovine serum albumin (BIOFROXX, Germany). After rinsing thrice with TBST, the membrane was incubated with the respective secondary antibodies for 1 h at room temperature and washed again with TBST. An enhanced chemiluminescence solution was used for the visualization of the bands (Thermo Fisher Scientific). Finally, relative grey density values of the protein bands were quantified using ImageJ 1.45 software and normalized to the density of actin. The specific primary antibodies used were anti-β-actin (A1978, Sigma-Aldrich, 1:50,000), anti-Bif-1 (4467S, CST, United States, 1:1,000), anti-NF-κB p65 (6956S, CST, United States, 1:2,000), anti-Phospho-NF-κB p65 (3033S, CST, United States, 1:2000), anti-NLRP3 (15101S, CST, United States, 1:1,000), anti-Caspase-1 (24232S, CST, United States, 1:2,000), anti-ASC/TMS1 (67824S, CST, United States, 1:2,000), anti-IL-18 (57058S, CST, United States, 1:1,000), anti-IL-1β (12242S, CST, United States, 1:1,000), anti-p62 (88588S, CST, United States, 1:2,000), anti-ULK1 (8054S, CST, United States, 1:1,000), anti-Phospho-ULK1 (6888T, CST, United States, 1:2,000), anti-AMPKα (2532S, CST, United States, 1:1,000), anti-Phospho-AMPKα (2535S, CST, United States, 1:1,000), and anti-LC3B (A19665, Abclonal, China, 1:2,000). The secondary antibodies used were goat polyclonal antibody to rabbit IgG H&L HRP (CST, United States, 1:5,000) or goat polyclonal antibody to mouse IgG H&L HRP (CST, United States, 1:5,000).

### ELISA

Supernatants from cell culture or mouse plasma were collected and stored at −80°C and then assayed for mouse IL-1β (KET7005, Abbkine, United States) and mouse IL-18 (202104, Jingmei Biotech, China) according to the manufacturer’s instructions.

### Quantitative Real-Time Polymerase Chain Reaction

Total RNA from J774A.1 cells and lung tissues was extracted using RNAiso Plus (TaKaRa Biotechnology) according to the manufacturer’s instructions, and the concentration was measured using a NanoDrop 2000 spectrophotometer (Thermo Fisher Scientific). cDNA was synthesized using Evo M-MLV RT Premix (Accurate Biology, China) according to the manufacturer’s instructions, and then quantitatively analyzed with SYBR Green Premix Pro Taq HS qPCR Kit (Accurate Biology) according to the manufacturer’s instructions. Relative gene expression was normalized to β-actin and calculated using the 2^−ΔΔCt^ method.

The sequences of primers used for real-time polymerase chain reaction (PCR) were: 5′-GCA​ACT​GTT​CCT​GAA​CTC​AAC​T-3′ and 5′-ATC​TTT​TGG​GGT​CCG​TCA​ACT-3′ for *Il1b*; 5′-GAC​TCT​TGC​GTC​AAC​TTC​AAG​G-3′ and 5′-CAG​GCT​GTC​TTT​TGT​CAA​CGA-3′ for *Il18*; 5′-ATT​ACC​CGC​CCG​AGA​AAG​G-3′ and 5′-TCG​CAG​CAA​AGA​TCC​ACA​CAG-3′ for *Nlrp3*, 5′-GGC​TGT​ATT​CCC​CTC​CAT​CG-3′ and 5′-CCA​GTT​GGT​AAC​AAT​GCC​ATG​T-3′ for *actin*.

### Lactate Dehydrogenase Release Assay

Culture supernatants were collected and assayed using a lactate dehydrogenase (LDH) cytotoxicity assay kit (KTB1110, Abbkine) according to the manufacturer’s instructions.

### Caspase-1 Activity Assay

Caspase-1 activity assay was conducted using a commercial Caspase-1 Assay Kit (KTA3020, Abbkine). The NLRP3 inhibitor MCC950 (10 μM, HY-12815A, MedChemExpress) and the caspase-1 inhibitor VRT-043198 (1 μM, HY-112226, MedChemExpress) were used as positive controls. Cell lysates were collected and assayed according to the instruction.

### Statistical Analysis

All data are presented as the mean ± SD. Statistical differences between the two groups were calculated using ANOVA followed by Tukey’s post-hoc test. Differences were considered significant at *p* < 0.05.

## Results

### Canagliflozin Inhibits NLRP3-Inflammasome Activation in LPS-Treated NIH Mice

**FIGURE 1:**
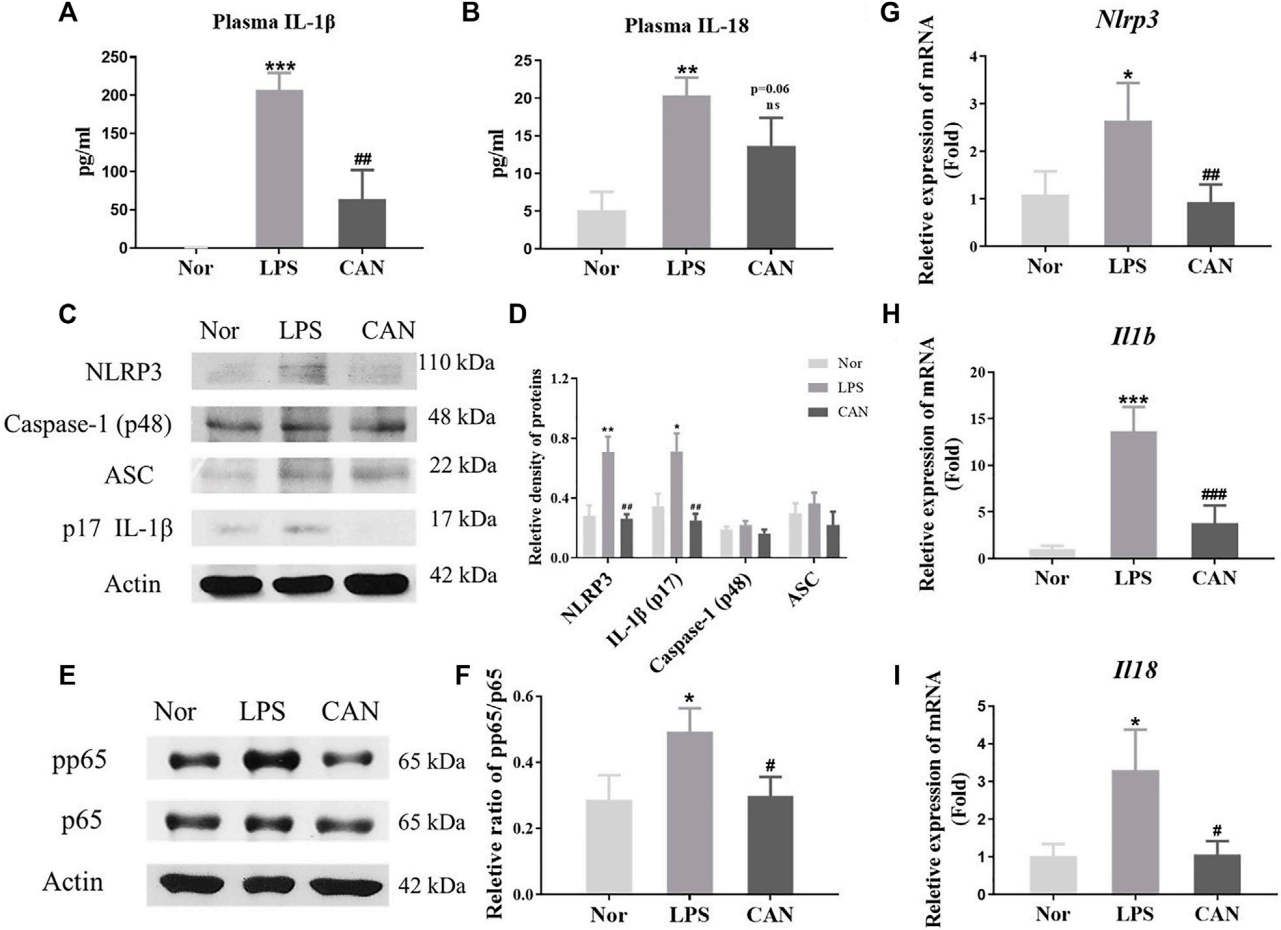
Canagliflozin inhibits NLRP3-inflammasome activation in LPS-treated NIH mice. CAN was administered intragastrically for 3 consecutive days. The normal and LPS groups were given the same amount of vehicle. After treatment, the mice were intraperitoneally injected with LPS (10 mg/kg). After 4 h of LPS induction, blood samples and lung tissues were collected for further assays. The plasma levels of **(A)** IL-1β and **(B)** IL-18 were measured by ELISA. Protein levels of **(C,D)** NLRP3, Caspase-1, ASC, IL-1β and **(E,F)** phosphorylation of p65 in lung tissues were measured using western blotting. The mRNA level of **(G)** NLRP3, **(H)** IL-1β and **(I)** IL-18 in lung tissues were quantified by qPCR. Nor, normal control group; LPS, untreated LPS control group; CAN, CAN-treated LPS group. Data were expressed as mean ± SD (*n* = 3). Differences with a probability value of <0.05 were considered significant (*p < 0.05, **p < 0.01, ***p < 0.001 vs. Nor; #*p* < 0.05, ##*p* < 0.01, ###*p* < 0.001 vs. LPS).

A previous study has shown that CAN exerts anti-inflammatory effects in LPS-treated NIH mice (Xu et al., 2018). However, the mechanism of this effect needs to be further elucidated. Several studies have shown that the NLRP3 inflammasome plays a key role in the occurrence and development of lung inflammation (Pinkerton et al., 2017; Freeman and Swartz, 2020; Paniri and Akhavan-Niaki, 2020). We examined the plasma levels of IL-1β and IL-18 and found that CAN reduced the levels of IL-1β and IL-18 (Figures 1A,B). Therefore, we speculate that CAN exerts anti-inflammatory effects by inhibiting the activation of NLRP3 inflammasomes. Subsequently, we used lung tissue for western blotting, and the data showed that CAN downregulated the levels of NLRP3 inflammasome-related proteins (Figures 1C,D). As NLRP3, IL-1β, and IL-18 are regulated by NF-κB (Bauernfeind et al., 2009; Zhu and Kanneganti, 2017; Cornut et al., 2020), we tested the influence of CAN on the NF-κB pathway. The results showed that CAN significantly inhibited the phosphorylation of p65 (Figures 1E,F). To further confirm this, we tested the effect of CAN on the transcription levels of NLRP3, IL-1β, and IL-18, and found that CAN significantly downregulated their mRNA levels (Figures 1G–I).

### CAN Inhibits NLRP3 Inflammasome Activation in LPS/ATP-Treated J774A.1 Cells

**FIGURE 2:**
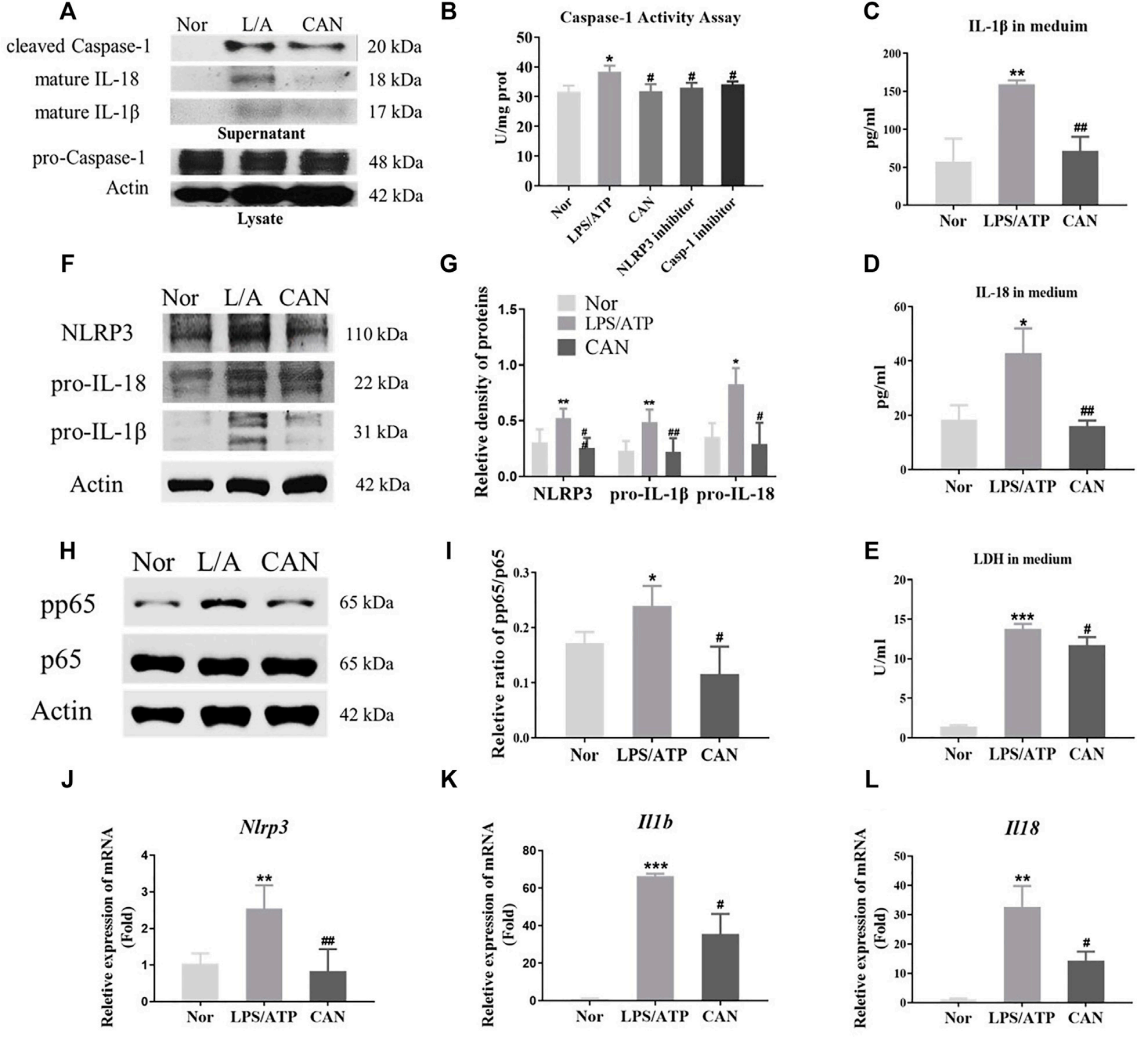
Canagliflozin inhibits NLRP3 inflammasome activation in LPS/ATP-treated J774A.1 cells. J774A.1 cells were primed with LPS (500 ng/ml) for 6 h in the presence or absence of CAN (10 μM), then stimulated with 5 mM ATP for 30 min. **(A)** Western blotting analysis of supernatant and whole cell lysate from J774A.1 cells. **(B)** Caspase-1 activity assay. The NLRP3 inhibitor MCC950 (10 μM) and the caspase-1 inhibitor VRT-043198 (1 μM) were used as positive controls. **(C)** IL-1β and **(D)** IL-18 in supernatant were measured by ELISA. **(E)** LDH release in supernatant was assessed. **(F,G)** Effects of CAN on the levels of NLRP3 inflammasome-associated proteins in J774A.1 cells. **(H,I)** CAN inhibited phosphorylation of p65 in J774A.1 cells. **(J–L)** CAN down-regulated mRNA levels of NLRP3, IL-1β and IL-18 in J774A.1 cells. Nor, normal control group; LPS/ATP (L/A), untreated LPS/ATP control group; CAN, CAN-treated LPS/ATP group. Data were expressed as mean ± SD (*n* = 3). Differences with a probability value of <0.05 were considered significant (*p < 0.05, **p < 0.01, ***p < 0.001 vs. Nor; #*p* < 0.05, ##*p* < 0.01, ###*p* < 0.001 vs. LPS/ATP).

To further confirm the above conclusions and study the underlying mechanism, we selected the mouse monocyte macrophage cell line J774A.1 for *in vitro* experiments. We investigated whether CAN has NLRP3 inflammasome-related anti-inflammatory activity *in vitro*. J774A.1 cells were primed with LPS (500 ng/ml) for 6 h in the presence or absence of CAN (10 µM) and stimulated with 5 mM ATP for 30 min. The supernatant was collected and the total protein or total RNA was extracted for further research. First, we found that CAN inhibited intracellular caspase-1 activity and reduced caspase-1 release in the supernatant, but did not affect intracellular pro-caspase-1 protein levels (Figures 2A,B). In addition, the release of IL-1β (Figures 2A–C), IL-18 (Figures 2A–D), and LDH (Figure 2E) in the supernatant was also decreased. Thereafter, whole cell lysate was analyzed using western blotting. The results showed that CAN significantly downregulated the protein levels of NLRP3, IL-1β, and IL-18 (Figures 2F,G) and inhibited the phosphorylation of p65 (Figures 2H,I). Finally, we conducted qPCR tests and the results showed that CAN downregulated the mRNA levels of NLRP3, IL-1β, and IL-18 (Figures 2J–L).

### CAN Inhibits NLRP3 Inflammasome Activation by Upregulating Bif-1

**FIGURE 3:**
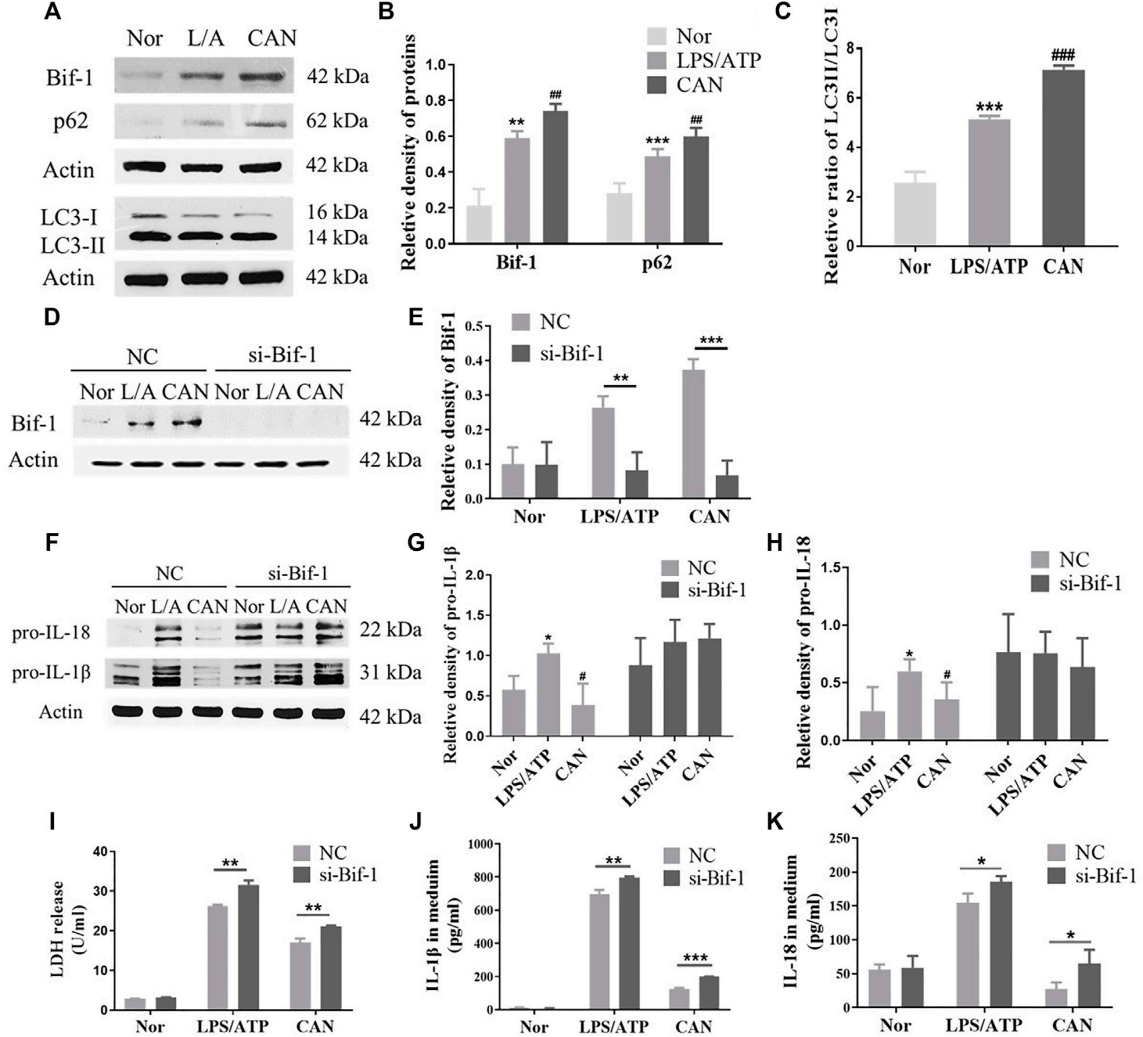
Canagliflozin inhibits NLRP3 inflammasome activation through up-regulating Bif-1. **(A–C)** Effects of CAN on Bif-1, p62 and the ratio of LC3-II/LC3-I. J774A.1 cells were transfected with Bif-1 siRNA or negative control siRNA using Lipofectamine 3000. Twenty-four hours after transfection, cells were primed with LPS (500 ng/ml) for 6 h in the presence or absence of CAN (10 μM), then stimulated with 5 mM ATP for 30 min. Whole cell lysis and supernatant were collected. Western blotting analysis of **(D,E)** Bif-1, **(F–H)** pro-IL-1β and pro-IL-18 were presented. **(I)** LDH release in supernatant was assessed. **(J,K)** ELISA of IL-1β and IL-18 in supernatant. Nor, normal control group; LPS/ATP (L/A), untreated LPS/ATP control group; CAN, CAN-treated LPS/ATP group. NC, negative control group; si-Bif-1, group treated with siRNA of Bif-1. Data were expressed as mean ± SD (*n* = 3). Differences with a probability value of <0.05 were considered significant. **(B,C,G,H)** *p < 0.05, **p < 0.01, ***p < 0.001 vs Nor; #*p* < 0.05, ##*p* < 0.01, ###*p* < 0.001 vs L/A. **(E)** and **(I–K)** *p < 0.05, **p < 0.01, ***p < 0.001.

Autophagy is an important mechanism by which cells degrade damaged proteins and organelles (Ma et al., 2013). In addition, several studies have shown that autophagy has a significant impact on the immune regulation of immune cells (Liu et al., 2015; Cao et al., 2019; Qiu et al., 2019). Recently, an increasing number of studies have shown that autophagy can regulate the activation of NLRP3 inflammasome (Harris et al., 2011; Shi et al., 2012; Biasizzo and Kopitar-Jerala, 2020). Bif-1 plays an important role in autophagosome formation during early autophagy (Takahashi et al., 2007). It interacts with Beclin-1 through ultraviolet radiation resistance-associated gene and positively regulates PI3KC3 (Takahashi et al., 2007). CAN further upregulates the protein levels of Bif-1 (Figures 3A,B and Supplementary Figure 1) and p62 and the ratio of LC3-II/LC3-I (Figures 3A–C). This suggests that CAN may promote autophagy by upregulating Bif-1, thereby inhibiting NLRP3 inflammasome activation. To confirm this, knockdown of Bif-1 was performed using Bif-1-specific siRNA (Figures 3D,E). Twenty-four hours after transfection, cells were primed with LPS (500 ng/ml) for 6 h in the presence or absence of CAN (10 μM) and stimulated with 5 mM ATP for 30 min. Whole cell lysis and the supernatants were collected. Treatment with Bif-1 siRNA upregulated pro-IL-1β and pro-interleukin-18 and eliminated the effect of CAN (Figures 3F–H). In addition, the release of LDH, IL-1β, and IL-18 in the si-Bif-1 group was significantly increased compared to that in the NC group (Figures 3I–K).

### Bif-1 is Regulated by NF-κB and ULK1

**FIGURE 4:**
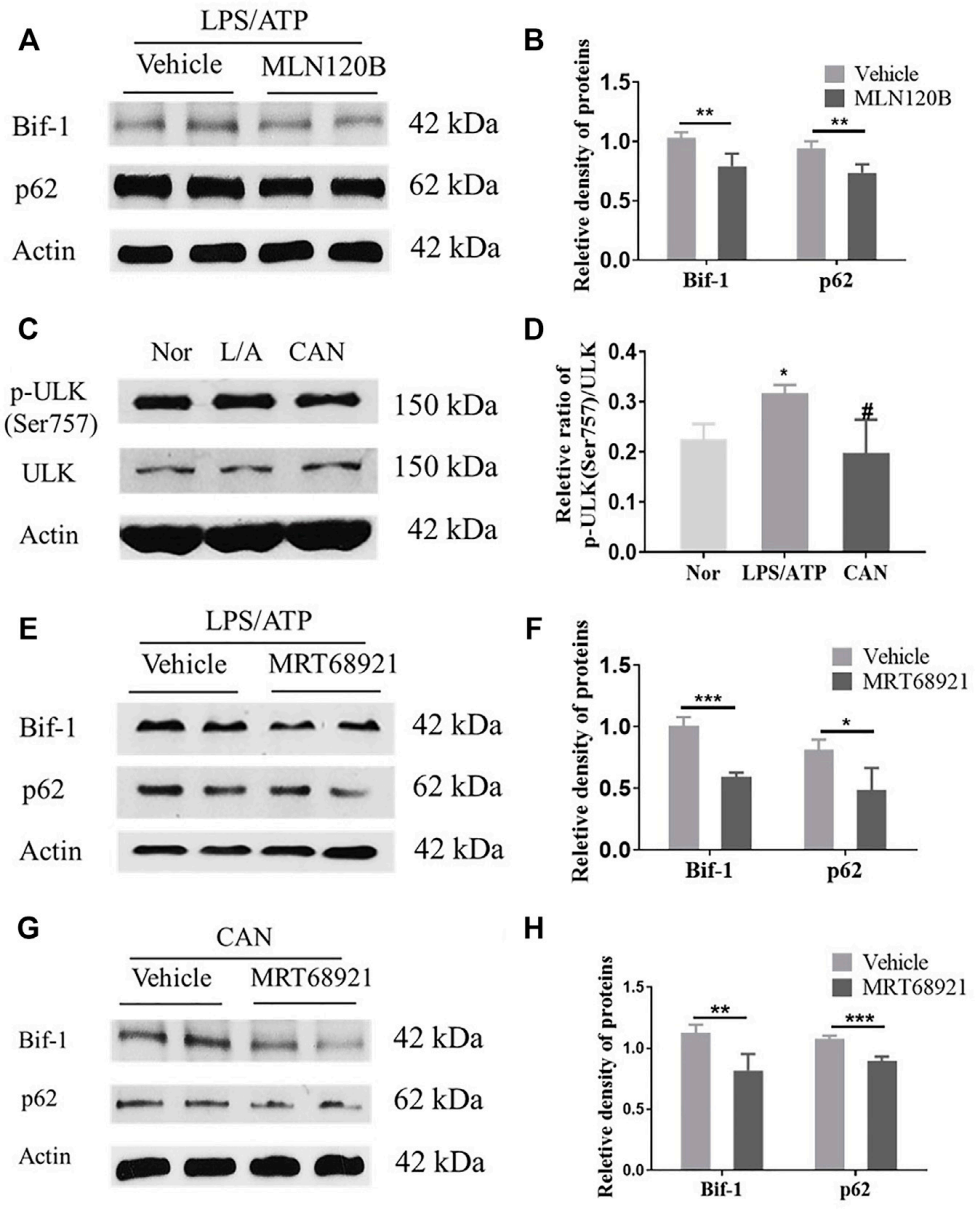
Bif-1 is regulated by NF-κB and ULK1. **(A,B)** J774A.1 cells were treated with LPS (500 ng/ml) for 6 h in the presence or absence of MLN120B (10 μM), then stimulated with 5 mM ATP for 30 min, protein levels of Bif-1 and p62 were analyzed. **(C,D)** CAN inhibits phosphorylation of ULK1 (Ser757). **(E,F)** J774A.1 cells were treated with LPS (500 ng/ml) for 6 h in the presence or absence of MRT68921 (1 μM), then stimulated with 5 mM ATP, protein levels of Bif-1 and p62 were analyzed. **(G,H)** J774A.1 cells were treated with LPS (500 ng/ml) and CAN (10 μM) for 6 h in the presence or absence of MRT68921 (1 μM), then stimulated with 5 mM ATP, protein levels of Bif-1 and p62 were analyzed. Nor, normal control group; LPS/ATP (L/A), untreated LPS/ATP control group; CAN, CAN-treated LPS/ATP group. Data were expressed as mean ± SD (*n* = 3). Differences with a probability value of <0.05 were considered significant. **(B,F,G)**, *p < 0.05, **p < 0.01, ***p < 0.001; **(D)** *p < 0.05 vs. Nor; #*p* < 0.05 vs. LPS/ATP.

As Bif-1 was significantly upregulated (Figures 3A,B) after LPS stimulation, we speculated that Bif-1 may be regulated by the NF-κB pathway. We used MLN120B, which inhibits the phosphorylation of IKKβ, to verify this hypothesis. J774A.1 cells were treated with LPS/ATP in the presence or absence of MLN120B (10 μM), and the protein levels of Bif-1 and p62 in the MNL120B-treated group were significantly downregulated compared to the vehicle group (Figures 4A,B).

After CAN treatment, Bif-1 was further upregulated compared with the LPS/ATP group (Figures 3A,B). However, as CAN inhibited the activation of NF-κB (Figures 2G,H), it may upregulate Bif-1 in some other manner. The serine/threonine-protein kinase ULK1 (ULK1) is essential for the initial stages of autophagy (Petherick et al., 2015). Under conditions of nutritional deficiency, AMP-activated protein kinase (AMPK) phosphorylates the Ser 317 and Ser 777 sites of ULK1 to activate autophagy. Additionally, under conditions of adequate nutrition, the mammalian target of rapamycin (mTOR) can phosphorylate the Ser 757 site of ULK1 to block its interaction with AMPK, thereby deactivating ULK1 (Kim et al., 2011). Herein, we found that CAN significantly inhibited the phosphorylation of ULK1 (Ser 757) (Figures 4C,D). Therefore, we speculate that CAN may regulate Bif-1 through ULK1. A ULK1 inhibitor, MRT68921, was used to test this hypothesis. J774A.1 cells were treated with LPS/ATP alone or in combination with CAN in the presence or absence of MRT68921 (1 μM), and the protein levels of Bif-1 and p62 were analyzed. The results showed that in the LPS/ATP and CAN treatment groups, MRT68921 significantly downregulated Bif-1 and p62 (Figures 4E–H).

### CAN Inhibits Phosphorylation of p65 and ULK1 (Ser757) by Activating AMPK

**FIGURE 5:**
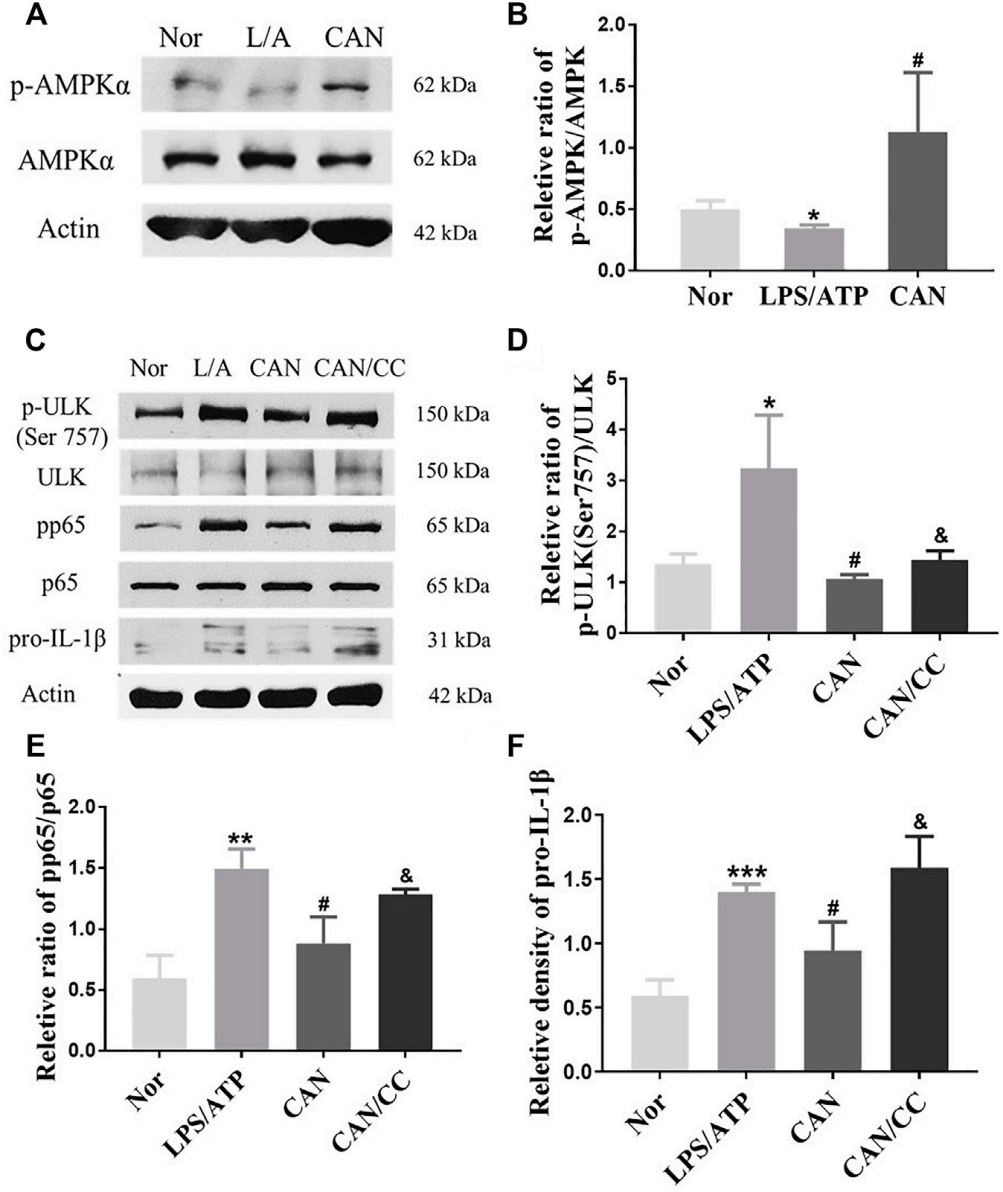
Canagliflozin inhibits phosphorylation of p65 and ULK1 (Ser 757) by activating AMPK. **(A,B)** CAN upregulated phosphorylation of AMPK. **(C–F)** J774A.1 cells were pretreated with Compound C (5 μM) for 1 h, then stimulated with LPS (500 ng/ml) in the presence or absence of CAN (10 μM) for 6 h, after that 5 mM ATP was added. Whole cell lysis was collected, phosphorylation of p65, ULK1 (Ser 757) and protein level of pro-IL-1β were assayed using western blotting. Nor, normal control group; LPS/ATP (L/A), untreated LPS/ATP control group; CAN, CAN-treated LPS/ATP group; CAN/CC, Compound C-treated CAN group. Data were expressed as mean ± SD (*n* = 3). Differences with a probability value of <0.05 were considered significant (*p < 0.05, **p < 0.01, ***p < 0.001 vs. Nor; #*p* < 0.05 vs. LPS/ATP; &*p* < 0.05 vs. CAN).

AMPK is involved in regulating cell metabolism and also plays an anti-inflammatory role in immune cells (O'Neill and Hardie, 2013). Several studies have shown that activation of AMPK can inhibit the phosphorylation of p65 (Chen et al., 2018; Xiang et al., 2019). Under starvation conditions, AMPK is activated, and mTORC1 is inhibited by AMPK through the phosphorylation of TSC2 and Raptor. Phosphorylation of ULK1 (Ser 757) is decreased, and ULK1 can subsequently interact with and is phosphorylated by AMPK on Ser 317 and Ser 777 (Kim et al., 2011). Our results showed that AMPK phosphorylation was significantly upregulated following CAN treatment (Figures 5A,B). Therefore, we speculated that CAN may affect the phosphorylation of p65 and ULK1 by activating AMPK. After treatment with Compound C (5 μM), an inhibitor of AMPK, phosphorylation of p65 and ULK1 (Ser 757) was upregulated and the protein level of pro-IL-1β was increased compared with that in the CAN group (Figures 5C–F).

## Discussion

CAN is a small-molecule hypoglycemic drug approved by the Food and Drug Administration and has been extensively studied because of its many new activities that are independent of its hypoglycemic effect. In our previous experiments, we found that CAN exerts anti-inflammatory effects both *in vivo* and *in vitro*. CAN significantly attenuated lipotoxicity and inflammation in cardiomyocytes (Sun et al., 2021). CAN significantly reduced IL-1β levels in the plasma and lung tissues of LPS-treated NIH mice and THP-1 cells stimulated by LPS (Xu et al., 2018; Niu et al., 2021). However, the exact molecular mechanisms remain to be elucidated. Given that the release of IL-1β is regulated by the NLRP3 inflammasome, we speculated that CAN may exert its anti-inflammatory activity by inhibiting the activation of the NLRP3 inflammasome. Our results showed that CAN downregulated the protein levels of NLRP3 inflammasome-associated proteins *in vivo* and *in vitro*, which may be due to its inhibition of p65 phosphorylation, which in turn leads to the downregulation of the transcription levels of these proteins.

Recent studies have indicated that SGLT2 inhibitors have anti-inflammatory activities by targeting the NLRP3 inflammasome (Yaribeygi et al., 2019; Pawlos et al., 2021). Empagliflozin inhibits NLRP3 inflammasomes, but the mechanism was likely associated with the regulation of ketones and insulin levels (Kim et al., 2020) or in a Ca^2+^-dependent manner (Byrne et al., 2020). Dapagliflozin was also found to inhibit the NLRP3 inflammasome, but the mechanism was likely mediated by activating AMPK (Ye et al., 2017). However, few studies have investigated the effect of CAN on the NLRP3 inflammasome. In this study, we found for the first time that CAN inhibits the activation of the NLRP3 inflammasome in a new way. CAN significantly inhibited the mRNA and protein levels of NLRP3. In addition, CAN also inhibited the activity of intracellular caspase-1 and reduced the release of caspase-1 in the supernatant. However, when NLRP3 was inhibited, the activity of caspase-1 was also downregulated. Therefore, whether the inhibition of caspase-1 activity by CAN is related to NLRP3 remains to be further investigated. Furthermore, we found that CAN significantly inhibited the release of IL-1β/18, which may be due to CAN inhibiting the production of its precursors and reducing the activity of caspase-1.

Several studies have shown that autophagy has a negative regulatory effect on the activation of NLRP3 inflammasomes (Harris et al., 2011; Shi et al., 2012; Biasizzo and Kopitar-Jerala, 2020). CAN has also been shown to promote autophagy in our previous study (Xu et al., 2018). We speculate that CAN may further inhibit the activation of NLRP3 inflammasomes by promoting autophagy. Bif-1 regulates the formation of autophagosomes and plays an important role in early stage autophagy (Takahashi et al., 2008). Our results indicate that CAN can inhibit the activation of the NLRP3 inflammasome by upregulating Bif-1. In immune cells, autophagy is considered a method of cell-autonomous control of inflammation (Deretic and Levine, 2018). Therefore, we speculated that the upregulation of Bif-1 after LPS stimulation may be a spontaneous balancing effect of macrophages on the inflammatory response and may be related to the activation of the NF-κB pathway. Our results preliminarily verify this conjecture, and more detailed mechanisms require further research. After CAN treatment, Bif-1 was further upregulated. However, as CAN inhibits the activation of NF-κB, it may upregulate Bif-1 in some other manner. ULK1 plays a key role in the initiation of early-stage autophagy. Our results indicate that CAN may regulate Bif-1 through ULK1, which requires further investigation.

Previous studies have indicated that activated AMPK can inhibit the NF-κB pathway (Salminen et al., 2011; Day et al., 2017). The NF-κB pathway directly mediates activation of the NLRP3 inflammasome (Bauernfeind et al., 2009; Qiao et al., 2012; McKee and Coll, 2020). Therefore, we speculated that AMPK activation by CAN might inhibit the activity of NLRP3 inflammasome by regulating the NF-κB pathway, but needs further validation. AMPK also regulates autophagy. mTORC1 is inhibited by activated AMPK through the phosphorylation of TSC2 and Raptor, phosphorylation of ULK1 (Ser 757) is decreased, and subsequently ULK1 can interact with and is phosphorylated by AMPK on Ser 317 and Ser 777 (Kim et al., 2011). Our results showed that AMPK was activated after CAN treatment. Therefore, we hypothesized that CAN can affect the phosphorylation of p65 and ULK1 by activating AMPK.

**FIGURE 6:**
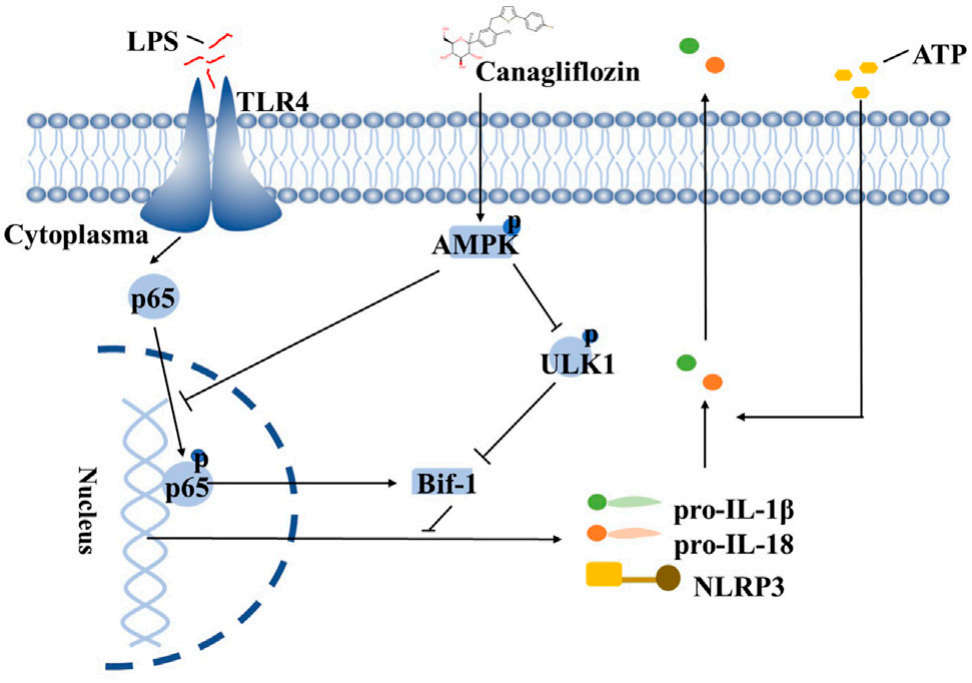
Schematic depicting that CAN ameliorates NLRP3 inflammasome-mediated inflammation through inhibiting NF-κB signaling and up-regulating Bif-1. The structure of CAN comes from DrugBank (https://go.drugbank.com/drugs/DB08907).

In summary, we have demonstrated that CAN significantly inhibited NLRP3 inflammasome activation. CAN inhibited the activation of the NF-κB pathway, thereby transcriptionally downregulating the levels of NLRP3 inflammasome-related proteins and inhibiting ULK1 (Ser 757) phosphorylation, thereby upregulating Bif-1 to promote early autophagy, both of which are mediated by the activation of AMPK (Figure 6). Diabetes is associated with an increased risk of pneumonia due to bacterial or viral infections (Shah and Hux, 2003; Hodgson et al., 2015). Excessive inflammasome activation and pro-inflammatory cytokine production are involved in the pathogenesis of respiratory diseases, and NLRP3 inflammasomes have been suggested as potential targets for such diseases. Our study is the first to report the inhibitory effect of CAN, a hypoglycemic drug, on the activation of NLRP3 inflammasomes in a non-hypoglycemic manner. We propose that Bif-1 regulates the activation of NLRP3 inflammasomes and is also involved in the anti-inflammatory mechanisms of CAN, which may provide new ideas for the treatment of patients with infectious pneumonia, particularly those concurrent with diabetes.

## Data Availability

The original contributions presented in the study are included in the article/Supplementary Material, further inquiries can be directed to the corresponding authors.
