# Supplementary material for: Canagliflozin Ameliorates NLRP3 Inflammasome-Mediated Inflammation Through Inhibiting NF-κB Signaling and Upregulating Bif-1
Source: Front Pharmacol. 2022 Mar 28;13:820541. doi: 10.3389/fphar.2022.820541 (PMC8996145; doi:10.3389/fphar.2022.820541)
Supplement: Supplementary file 1 [file Presentation1.PPTX]

## Slide 1
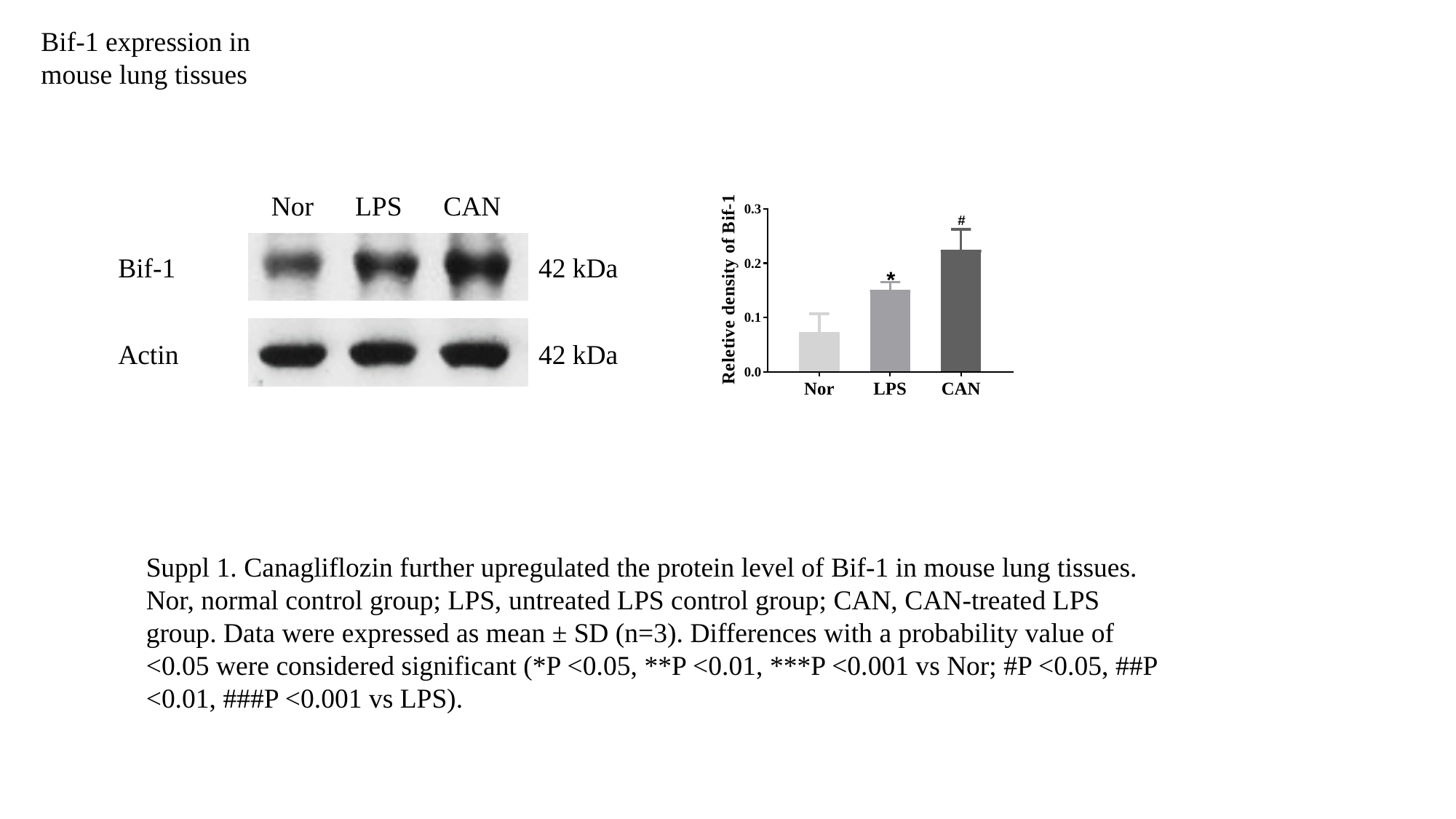

Bif-1 expression in mouse lung tissues
Nor LPS CAN
Bif-1
42 kDa
Actin
42 kDa
Suppl 1. Canagliflozin further upregulated the protein level of Bif-1 in mouse lung tissues. Nor, normal control group; LPS, untreated LPS control group; CAN, CAN-treated LPS group. Data were expressed as mean ± SD (n=3). Differences with a probability value of <0.05 were considered significant (*P <0.05, **P <0.01, ***P <0.001 vs Nor; #P <0.05, ##P <0.01, ###P <0.001 vs LPS).
