# Supplementary figures and images for: Canagliflozin Ameliorates NLRP3 Inflammasome-Mediated Inflammation Through Inhibiting NF-κB Signaling and Upregulating Bif-1
Source: Front Pharmacol. 2022 Mar 28;13:820541. doi: 10.3389/fphar.2022.820541 (PMC8996145; doi:10.3389/fphar.2022.820541)

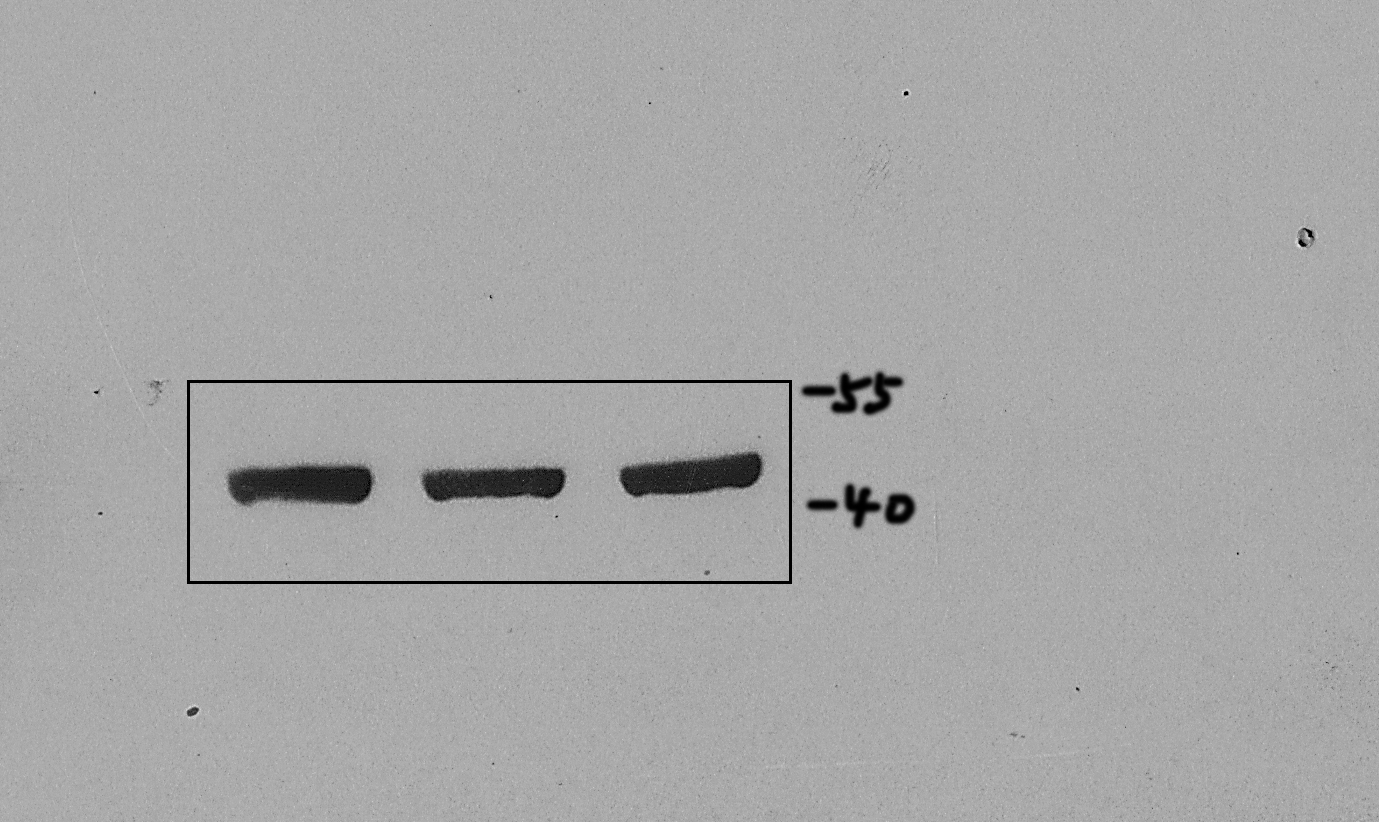

Supplement: Supplementary file 2 [file DataSheet1.ZIP › Fig 3/A/actin.TIF]

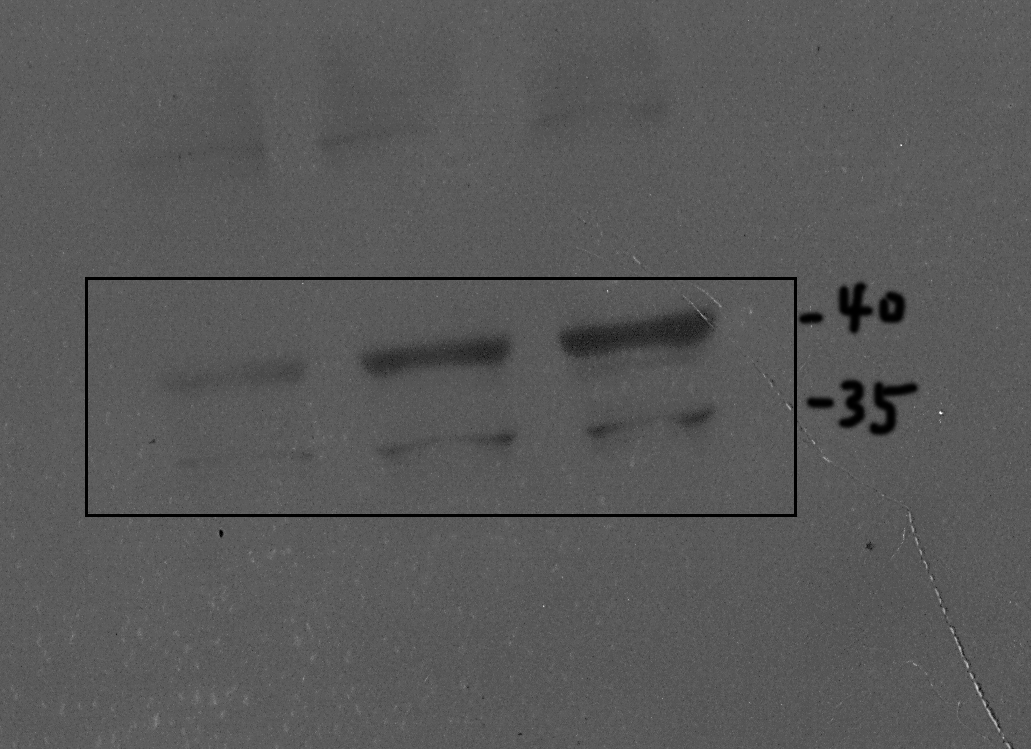

Supplement: Supplementary file 2 [file DataSheet1.ZIP › Fig 3/A/Bif-1.TIF]

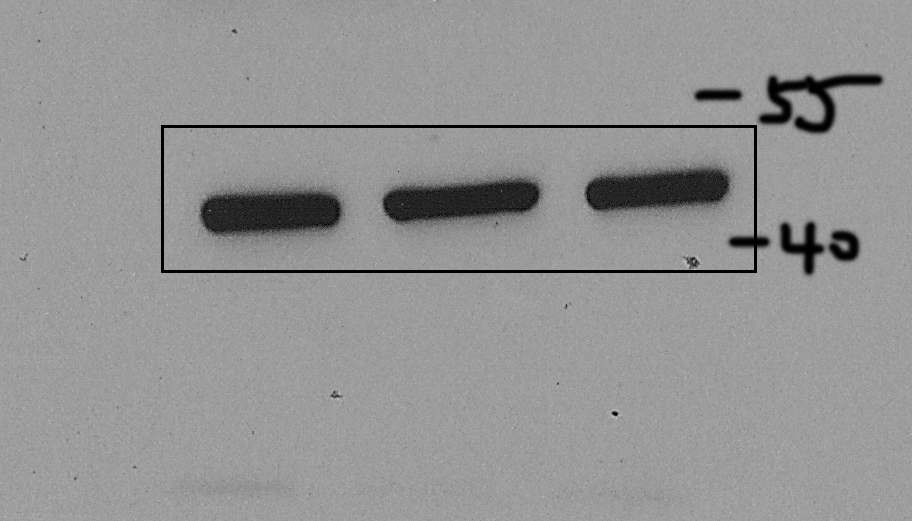

Supplement: Supplementary file 2 [file DataSheet1.ZIP › Fig 3/A/LC3-ACTIN.TIF]

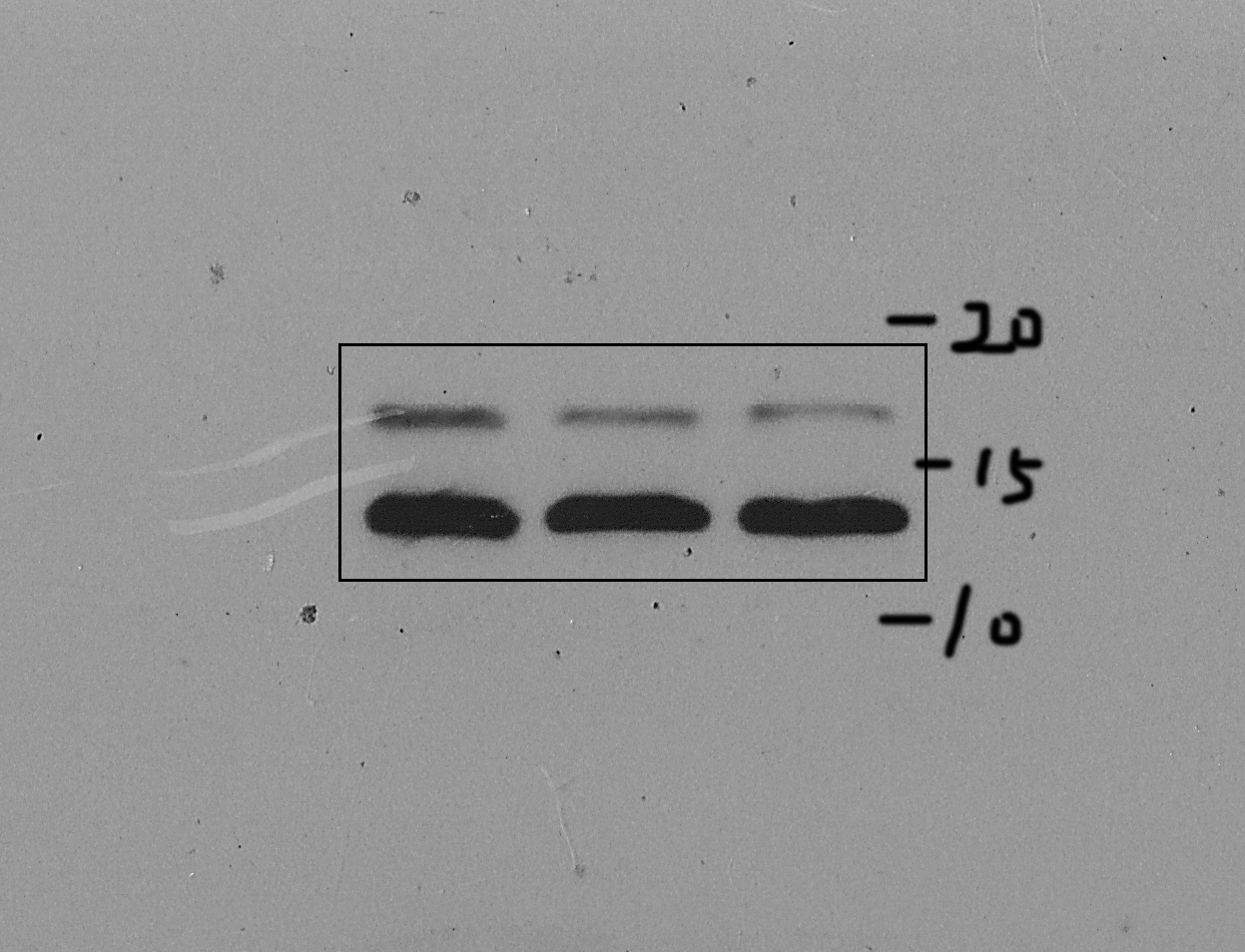

Supplement: Supplementary file 2 [file DataSheet1.ZIP › Fig 3/A/LC3.TIF]

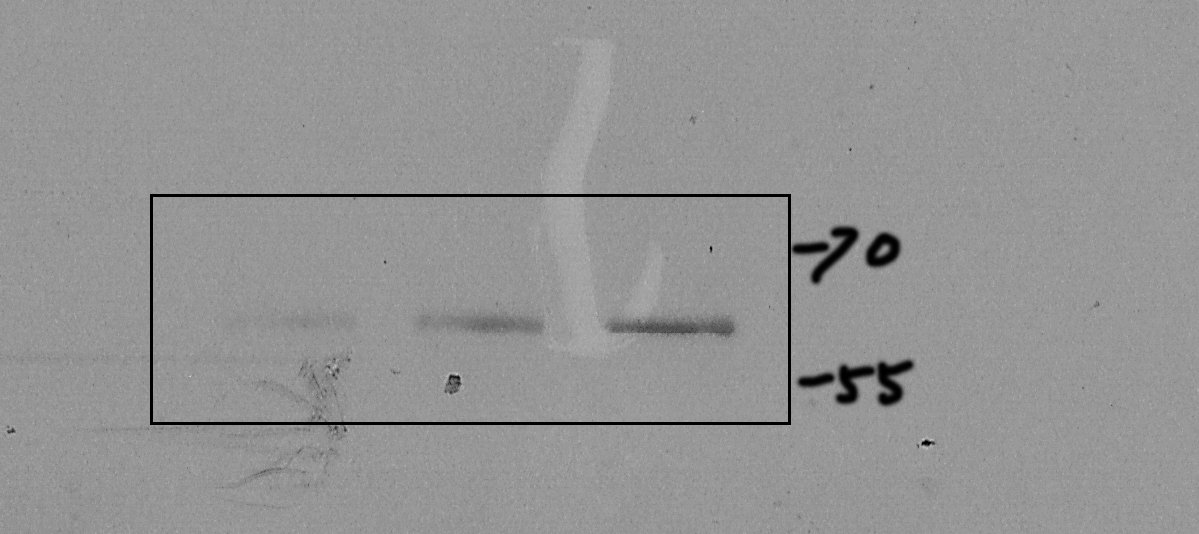

Supplement: Supplementary file 2 [file DataSheet1.ZIP › Fig 3/A/p62.TIF]

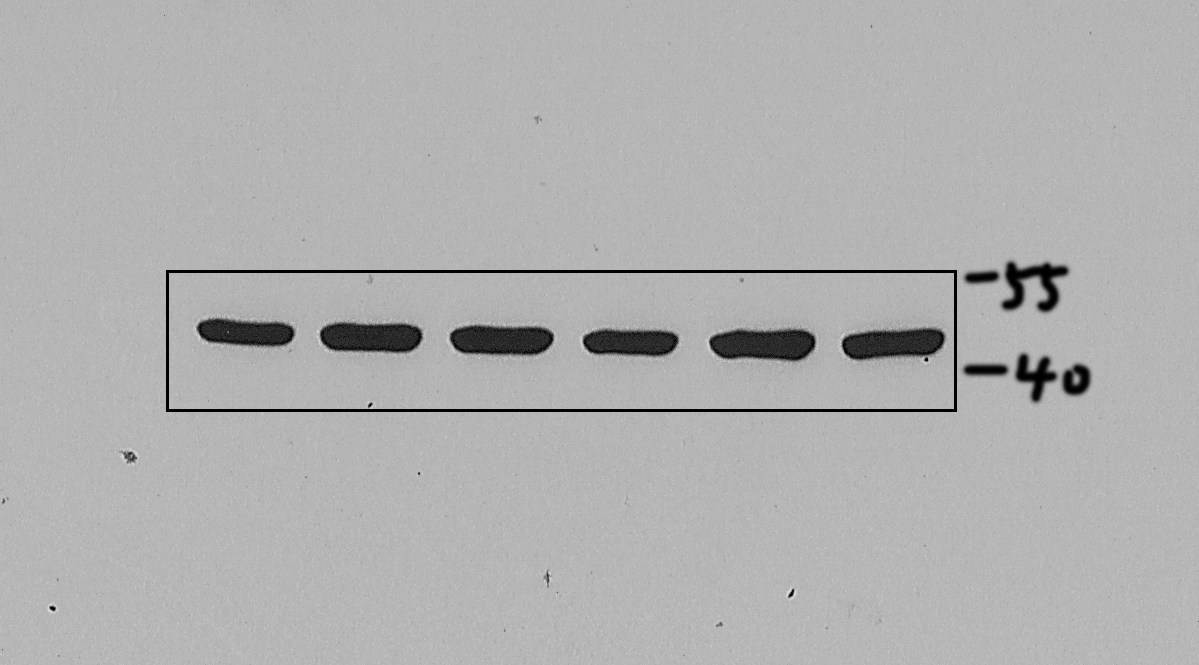

Supplement: Supplementary file 2 [file DataSheet1.ZIP › Fig 3/B/actin.TIF]

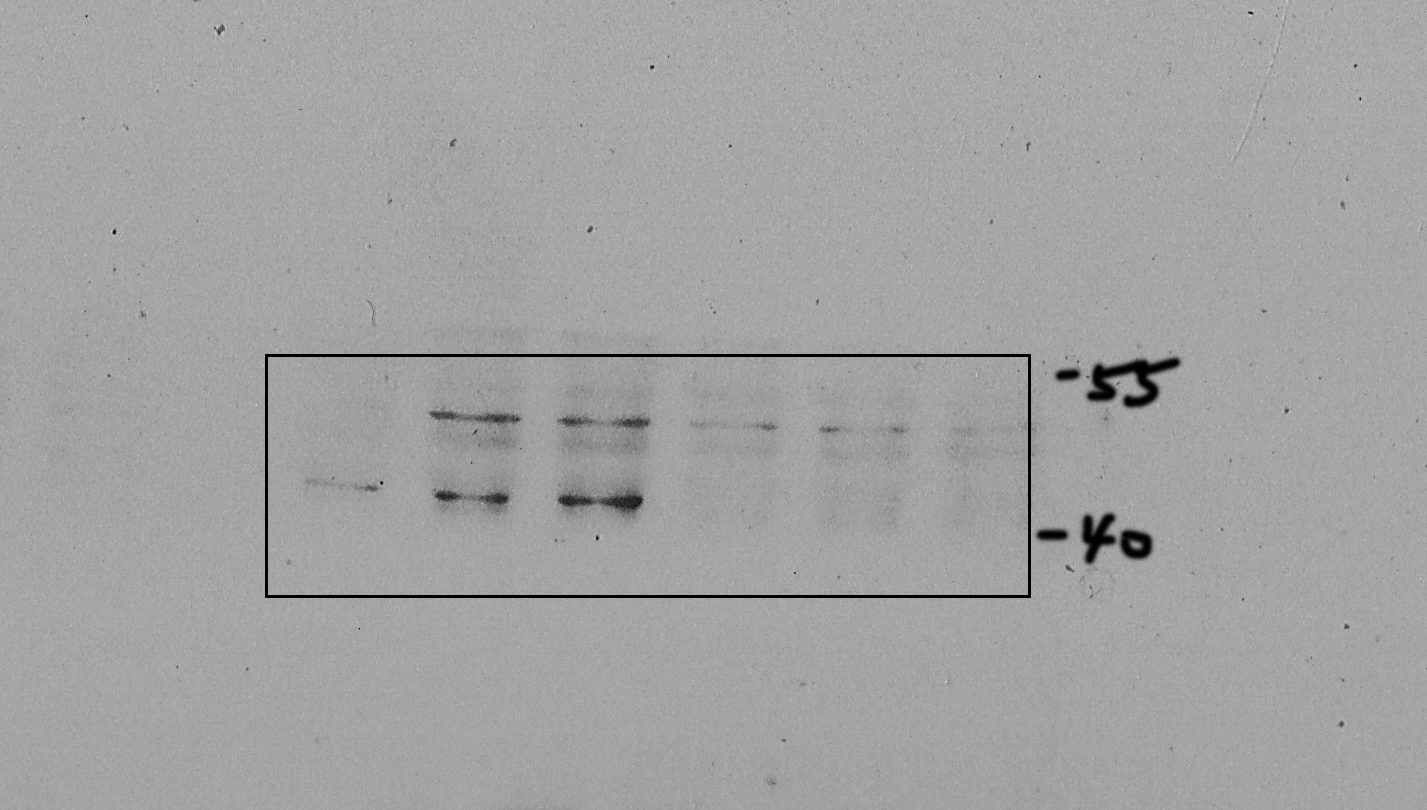

Supplement: Supplementary file 2 [file DataSheet1.ZIP › Fig 3/B/Bif-1.TIF]

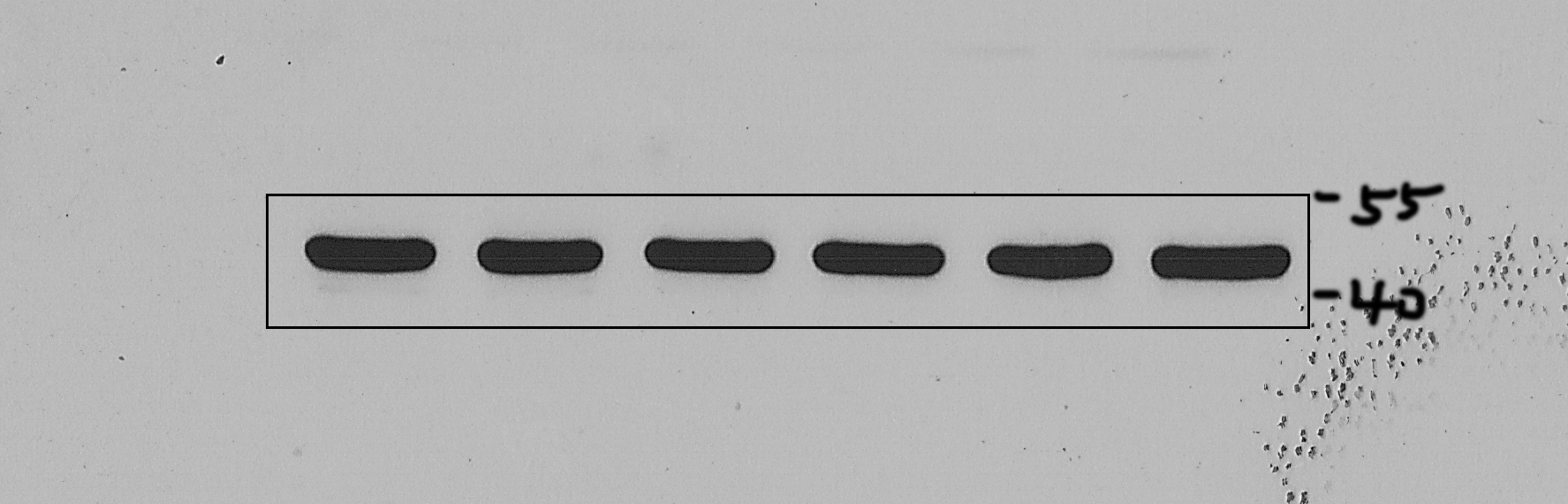

Supplement: Supplementary file 2 [file DataSheet1.ZIP › Fig 3/C/actin.TIF]

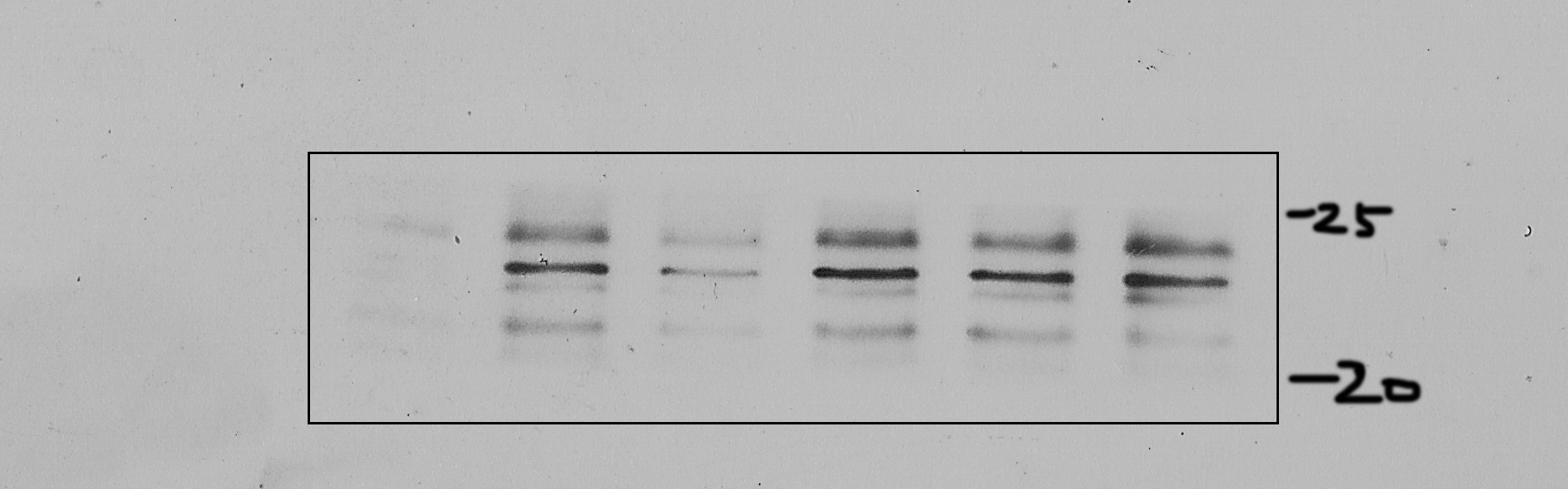

Supplement: Supplementary file 2 [file DataSheet1.ZIP › Fig 3/C/IL18.TIF]

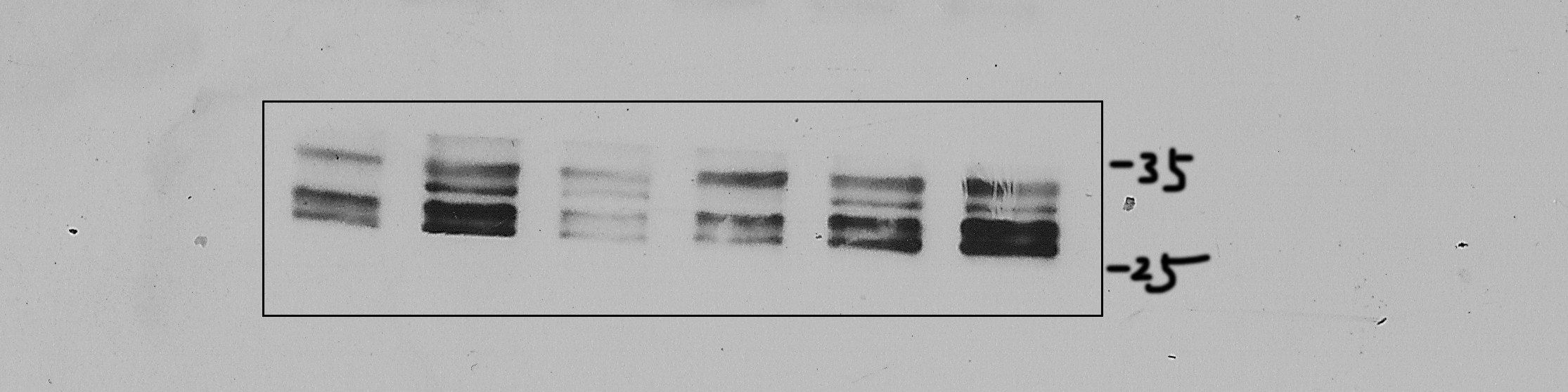

Supplement: Supplementary file 2 [file DataSheet1.ZIP › Fig 3/C/IL1beta.TIF]

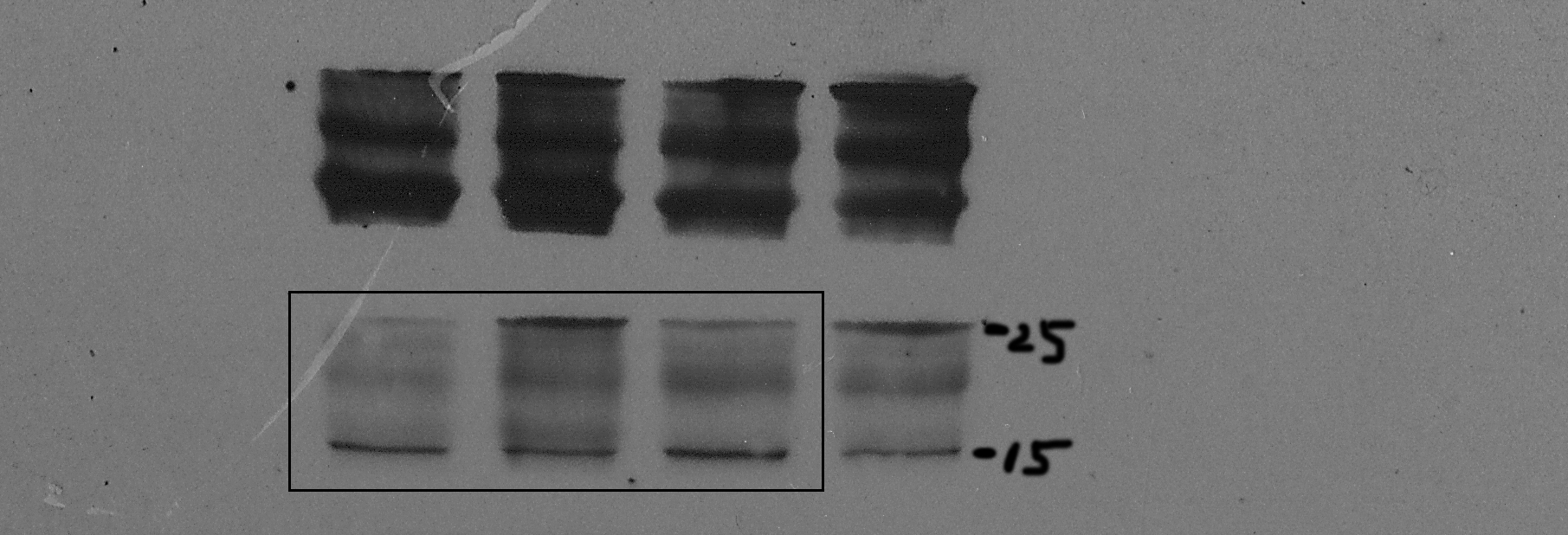

Supplement: Supplementary file 2 [file DataSheet1.ZIP › Fig 1/B/ASC.TIF]

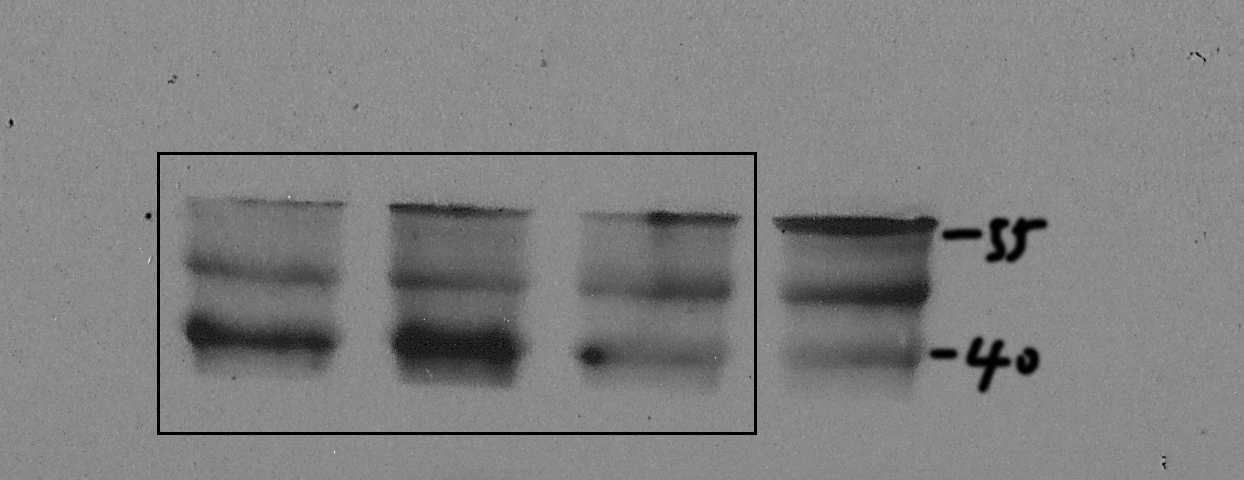

Supplement: Supplementary file 2 [file DataSheet1.ZIP › Fig 1/B/Caspase-1.TIF]

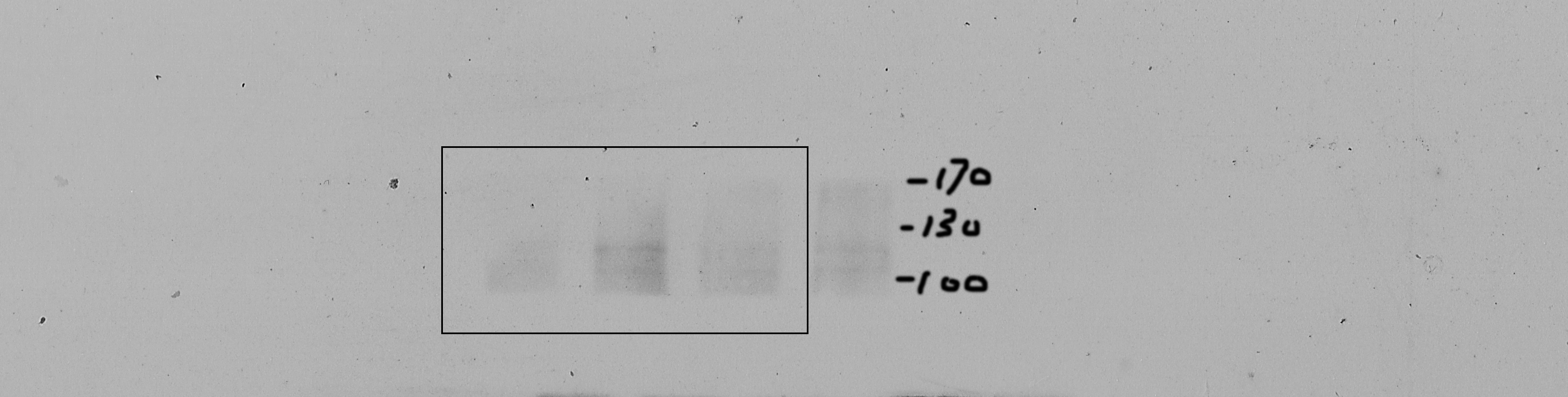

Supplement: Supplementary file 2 [file DataSheet1.ZIP › Fig 1/B/NLRP3.TIF]

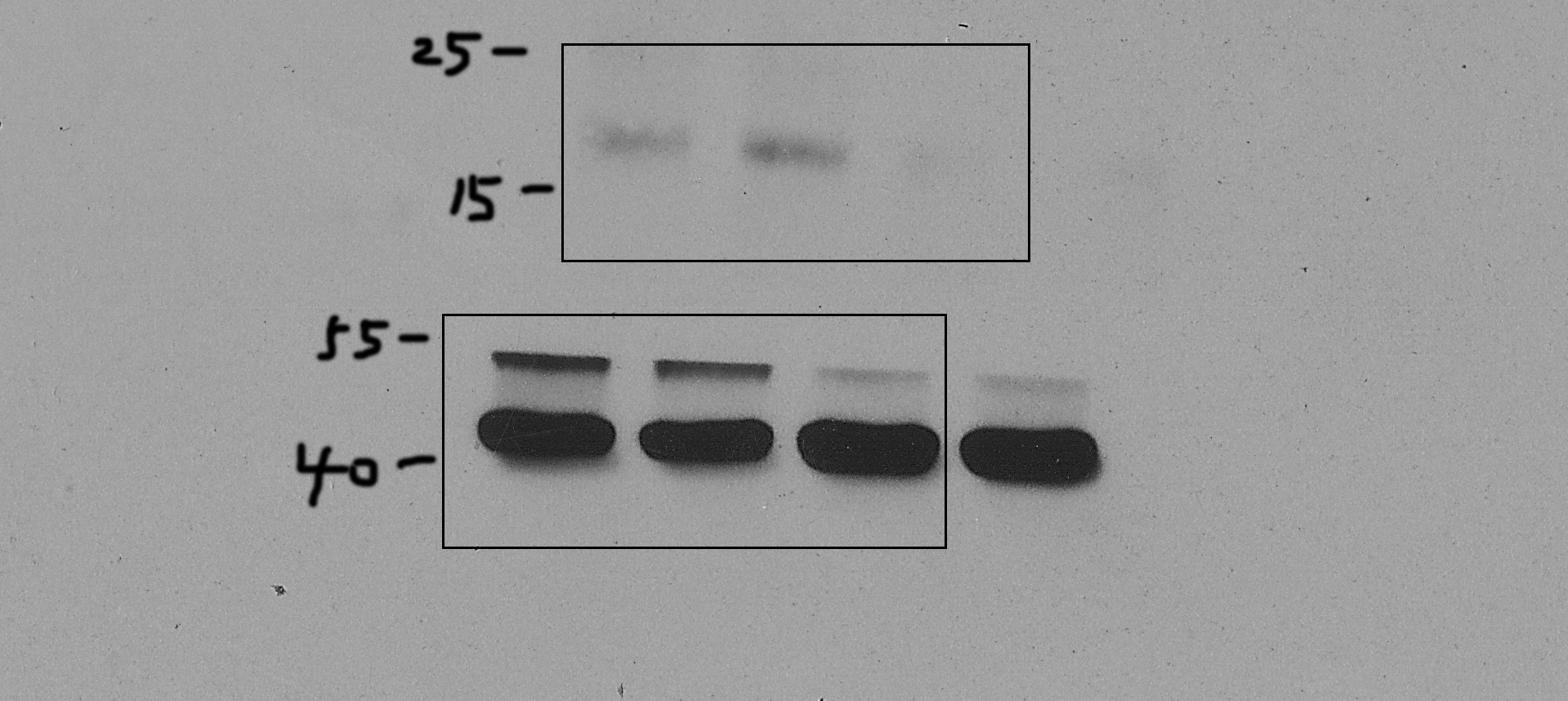

Supplement: Supplementary file 2 [file DataSheet1.ZIP › Fig 1/B/top-IL1beta bottom-actin.TIF]

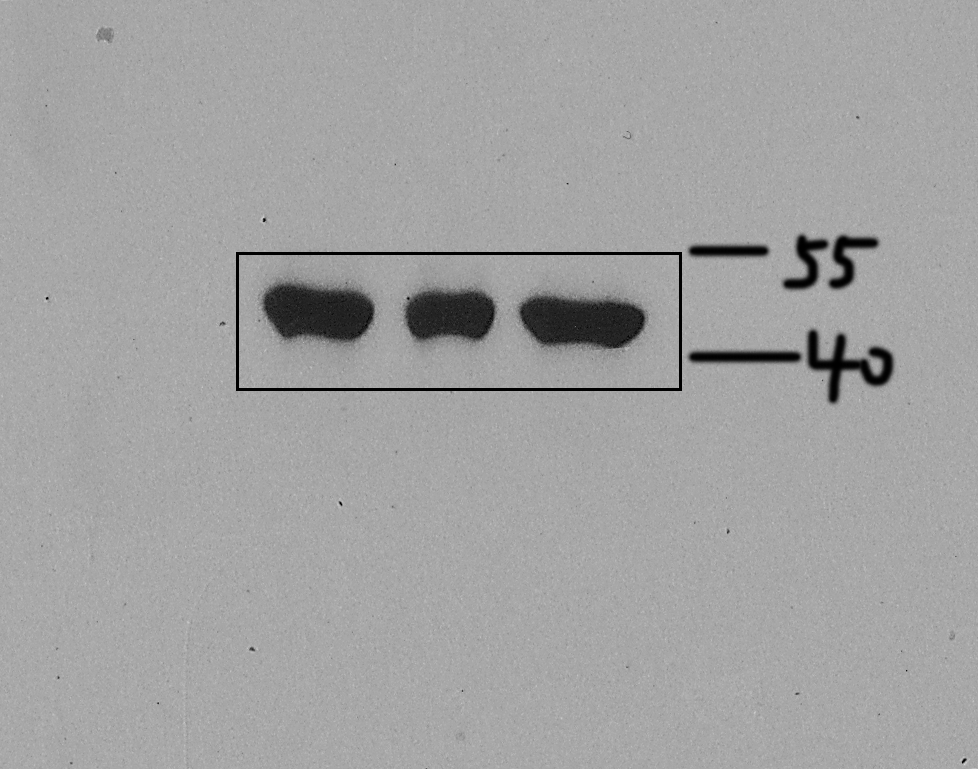

Supplement: Supplementary file 2 [file DataSheet1.ZIP › Fig 1/C/actin.TIF]

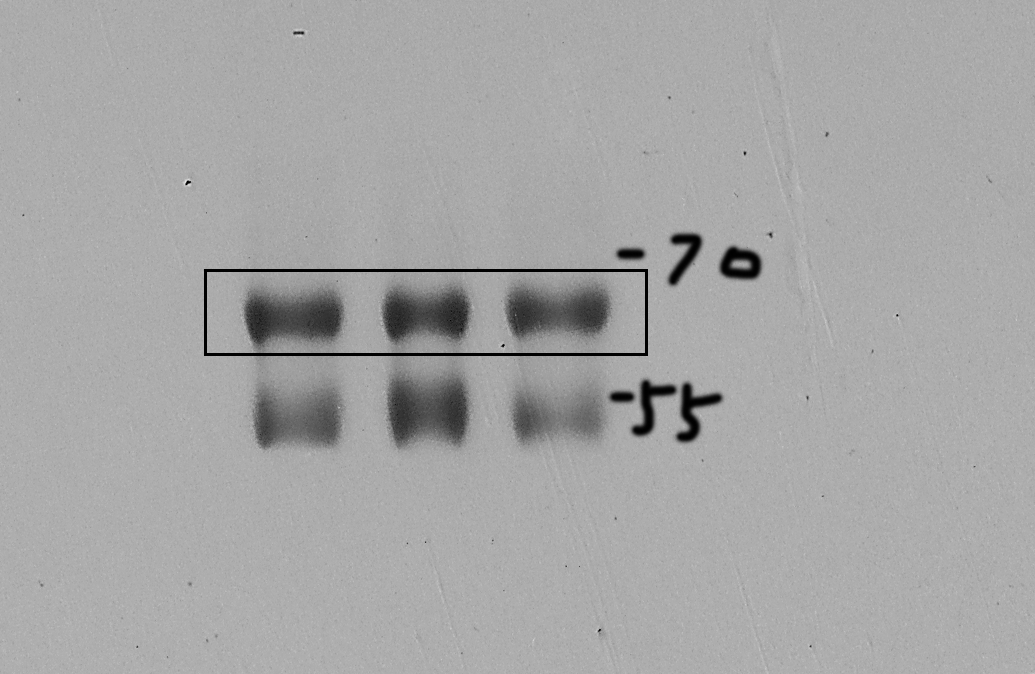

Supplement: Supplementary file 2 [file DataSheet1.ZIP › Fig 1/C/p65.TIF]

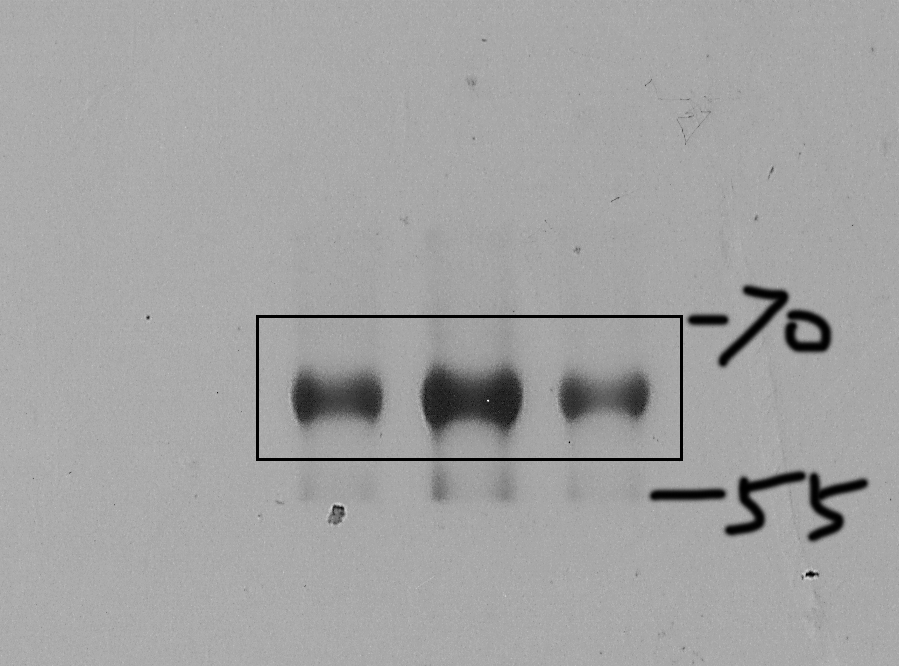

Supplement: Supplementary file 2 [file DataSheet1.ZIP › Fig 1/C/pp65.TIF]

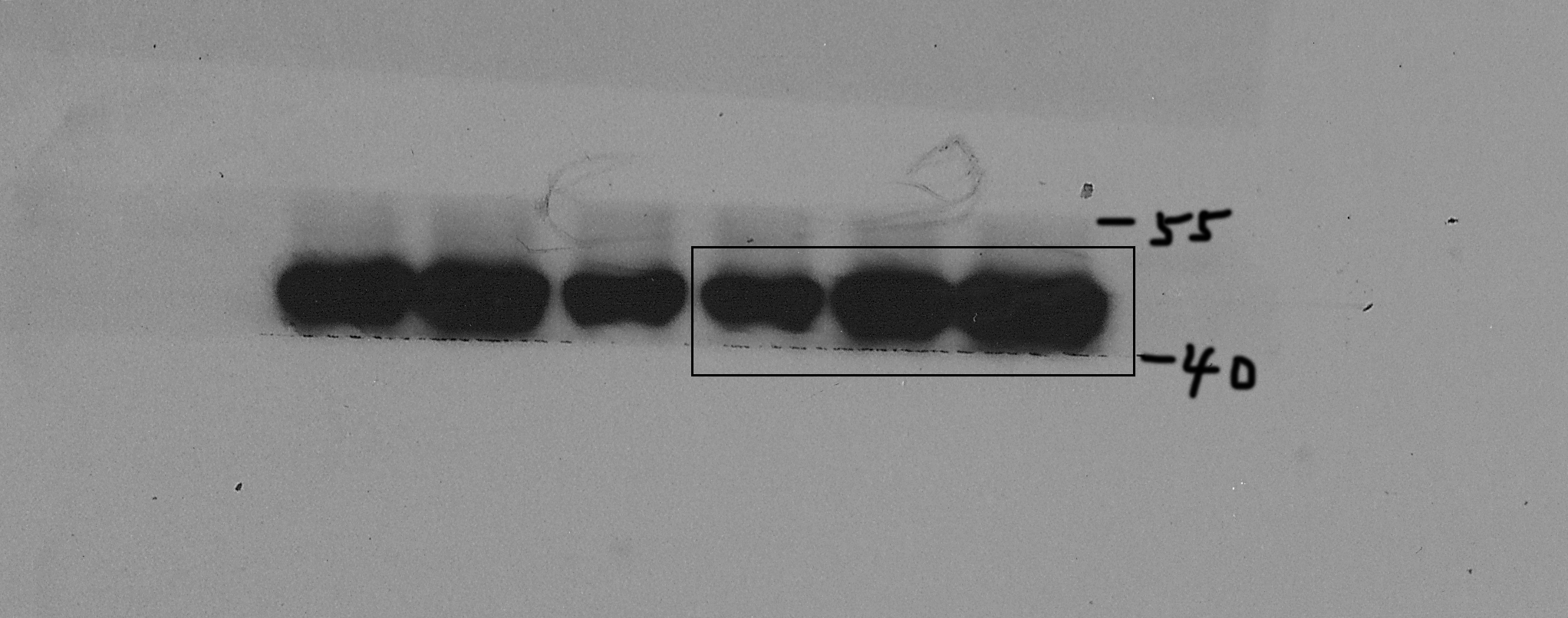

Supplement: Supplementary file 2 [file DataSheet1.ZIP › Fig 2/A/actin.TIF]

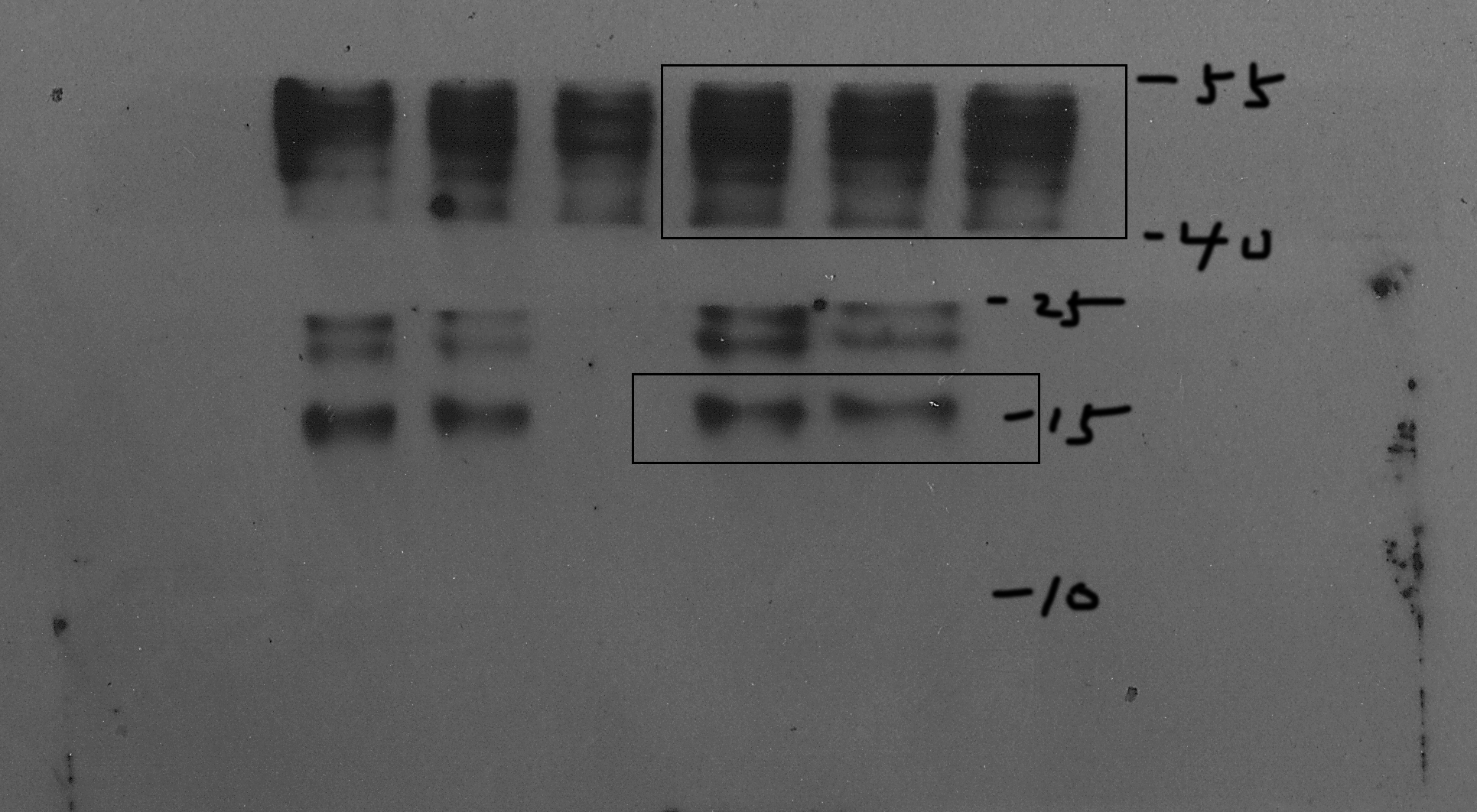

Supplement: Supplementary file 2 [file DataSheet1.ZIP › Fig 2/A/caspase-1.TIF]

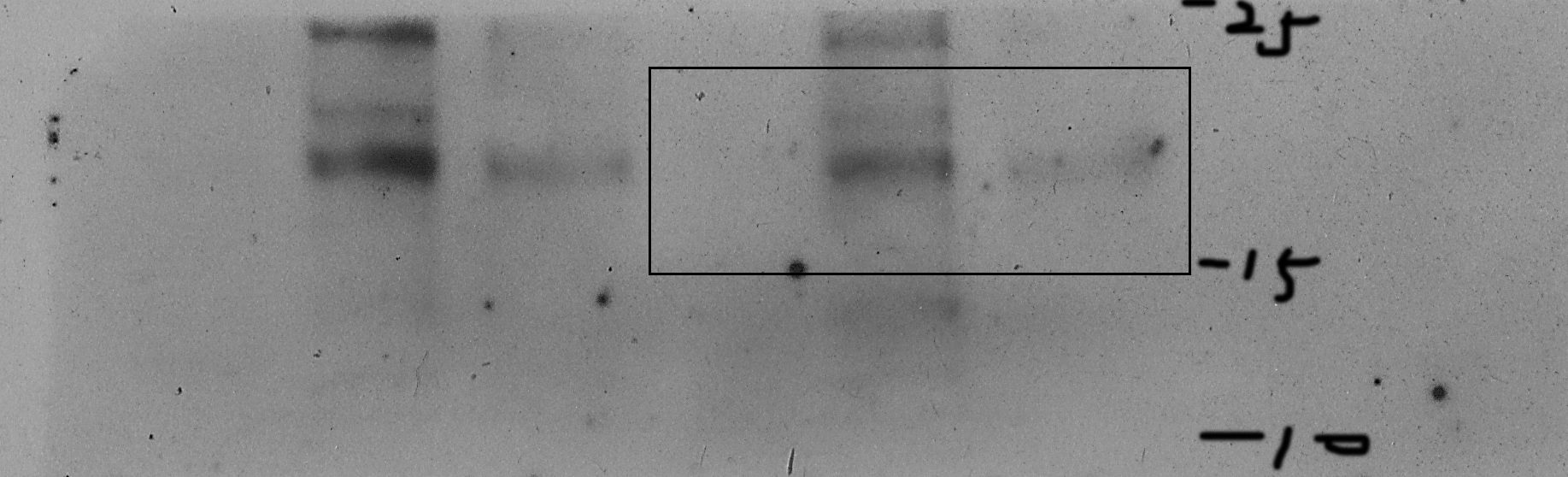

Supplement: Supplementary file 2 [file DataSheet1.ZIP › Fig 2/A/IL18.TIF]

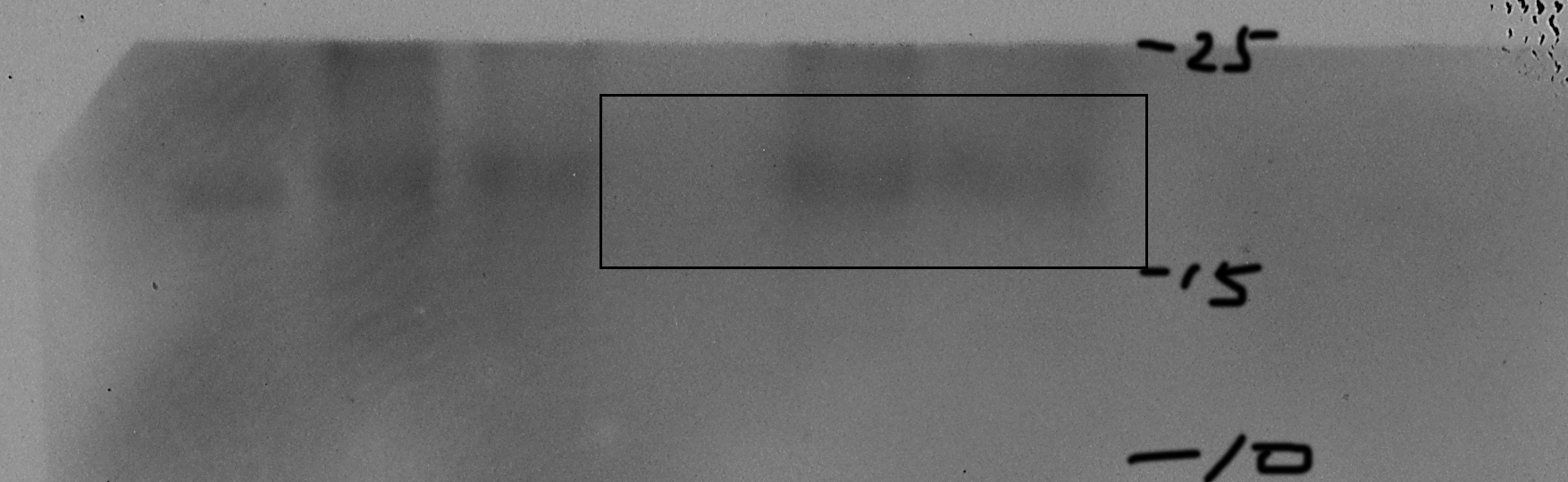

Supplement: Supplementary file 2 [file DataSheet1.ZIP › Fig 2/A/IL1B.TIF]

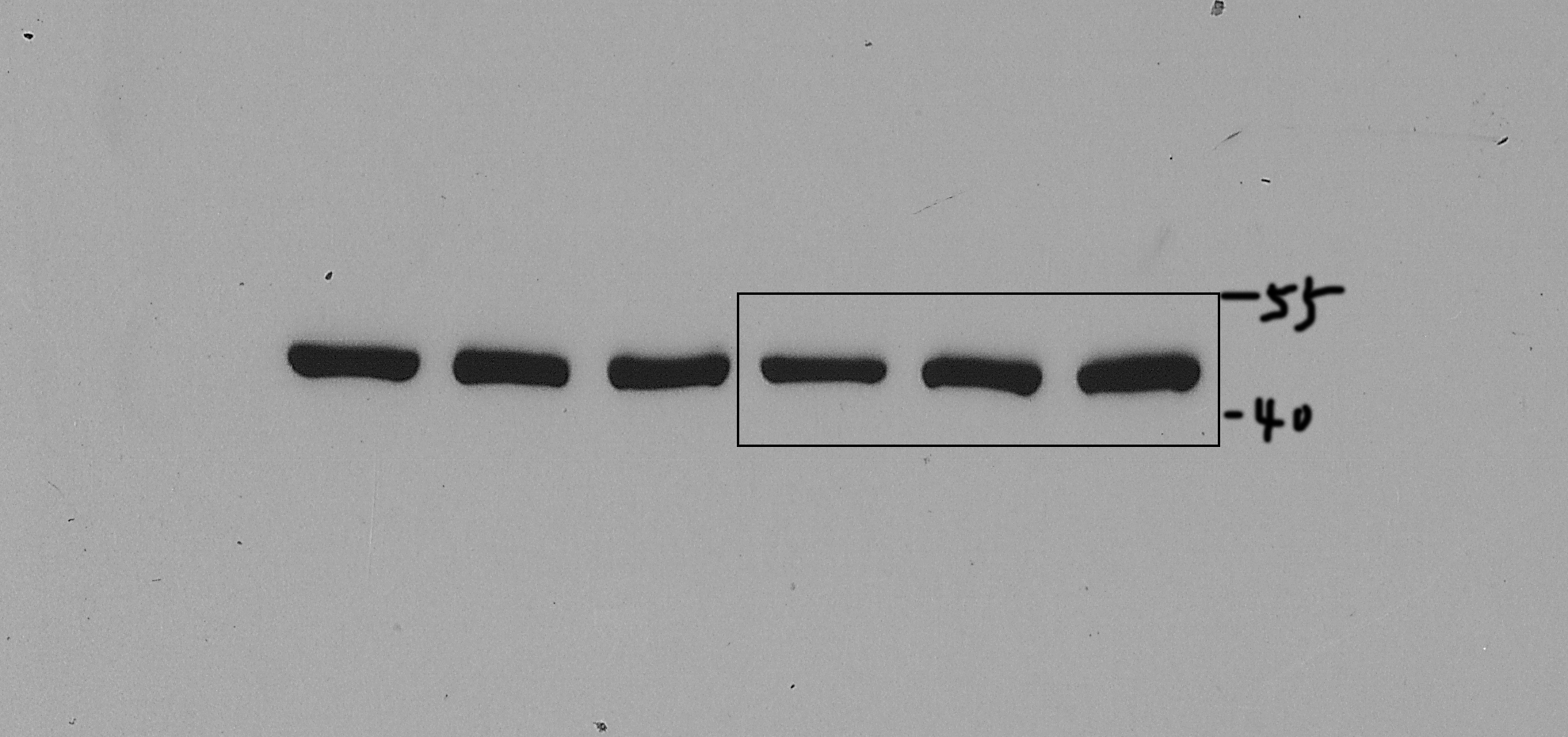

Supplement: Supplementary file 2 [file DataSheet1.ZIP › Fig 2/E/actin.TIF]

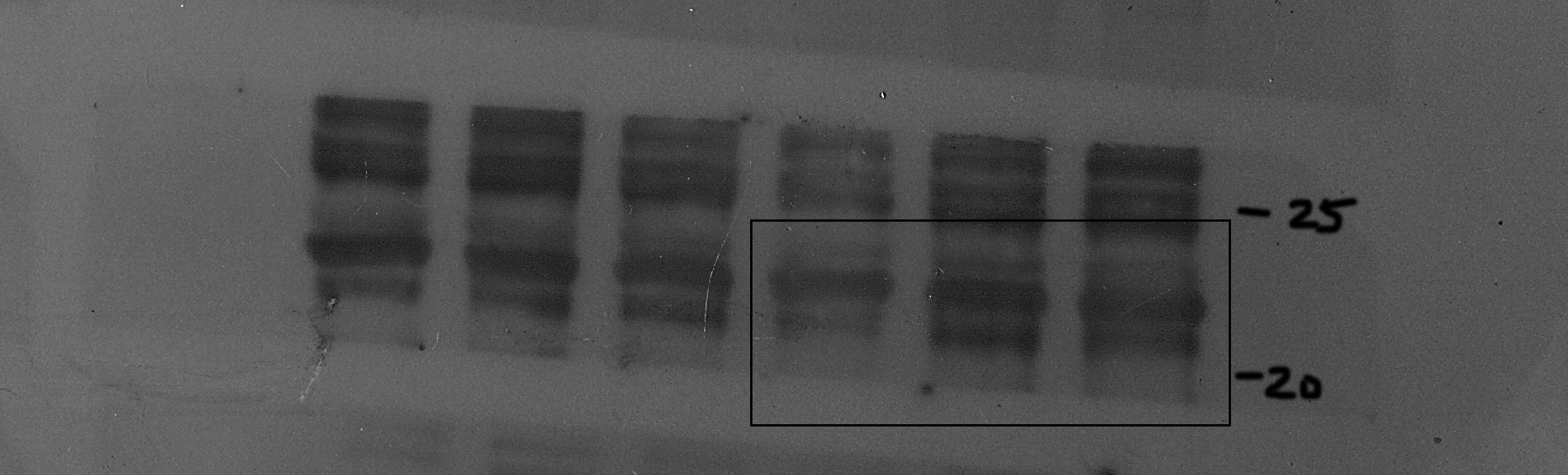

Supplement: Supplementary file 2 [file DataSheet1.ZIP › Fig 2/E/IL18.TIF]

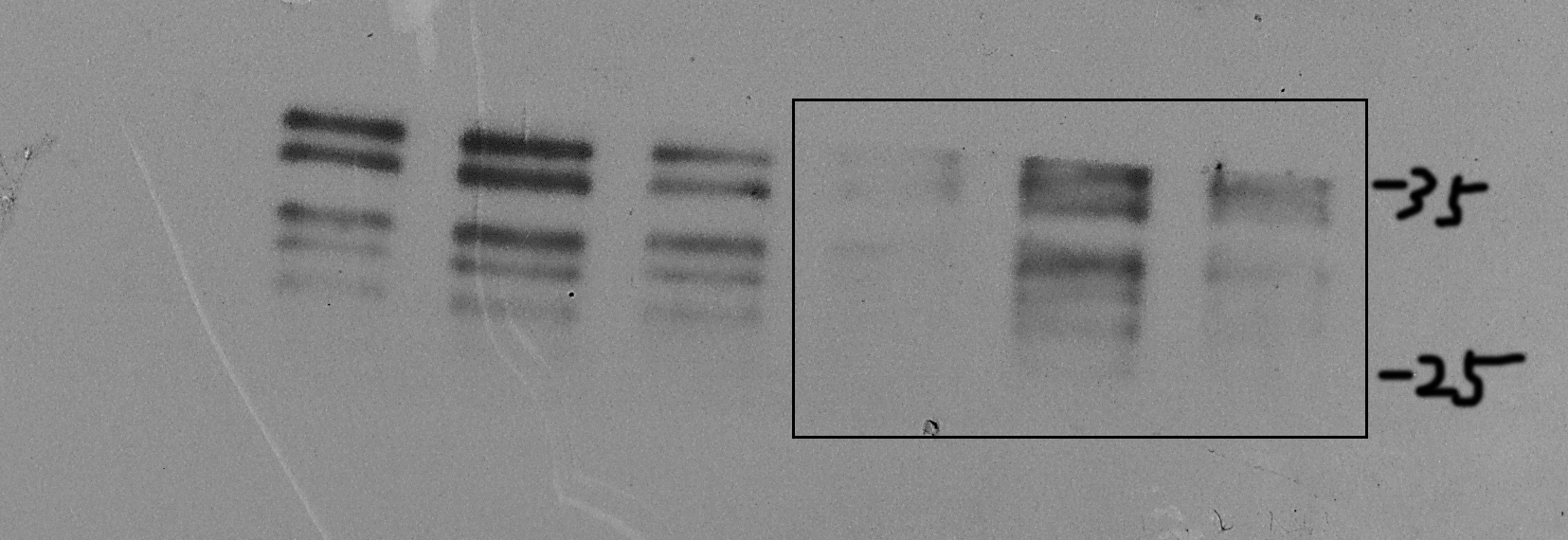

Supplement: Supplementary file 2 [file DataSheet1.ZIP › Fig 2/E/IL1beta.TIF]

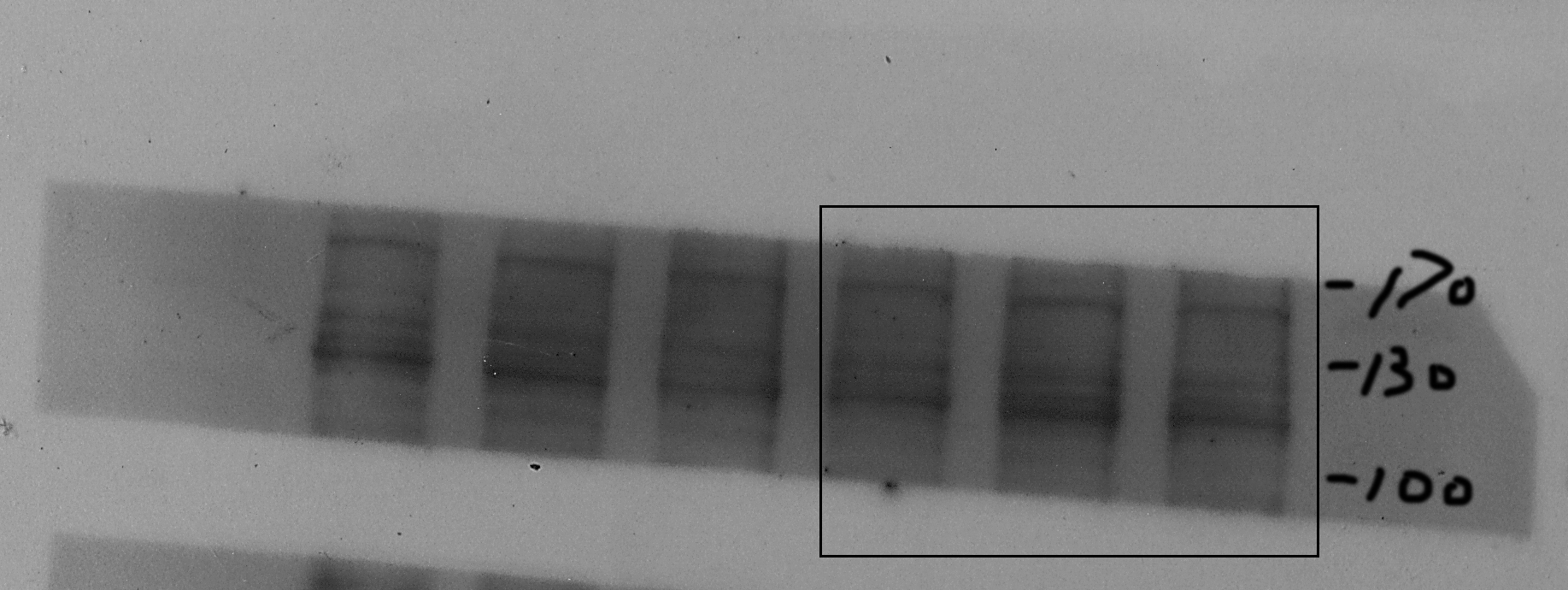

Supplement: Supplementary file 2 [file DataSheet1.ZIP › Fig 2/E/NLRP3.TIF]

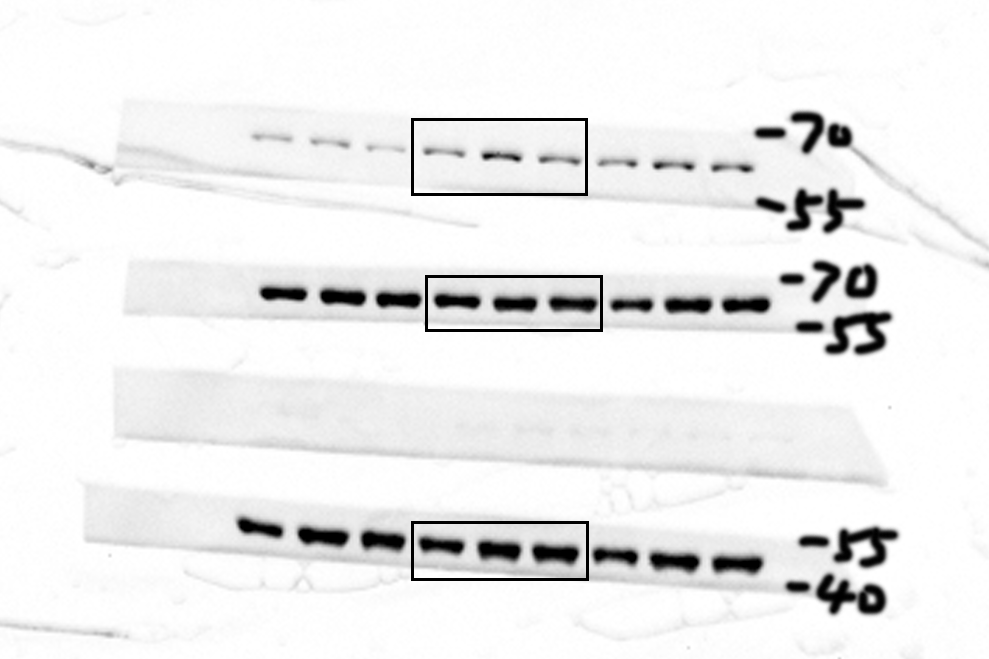

Supplement: Supplementary file 2 [file DataSheet1.ZIP › Fig 2/F/top-pp65 mid-p65 bottom-actin.tif]

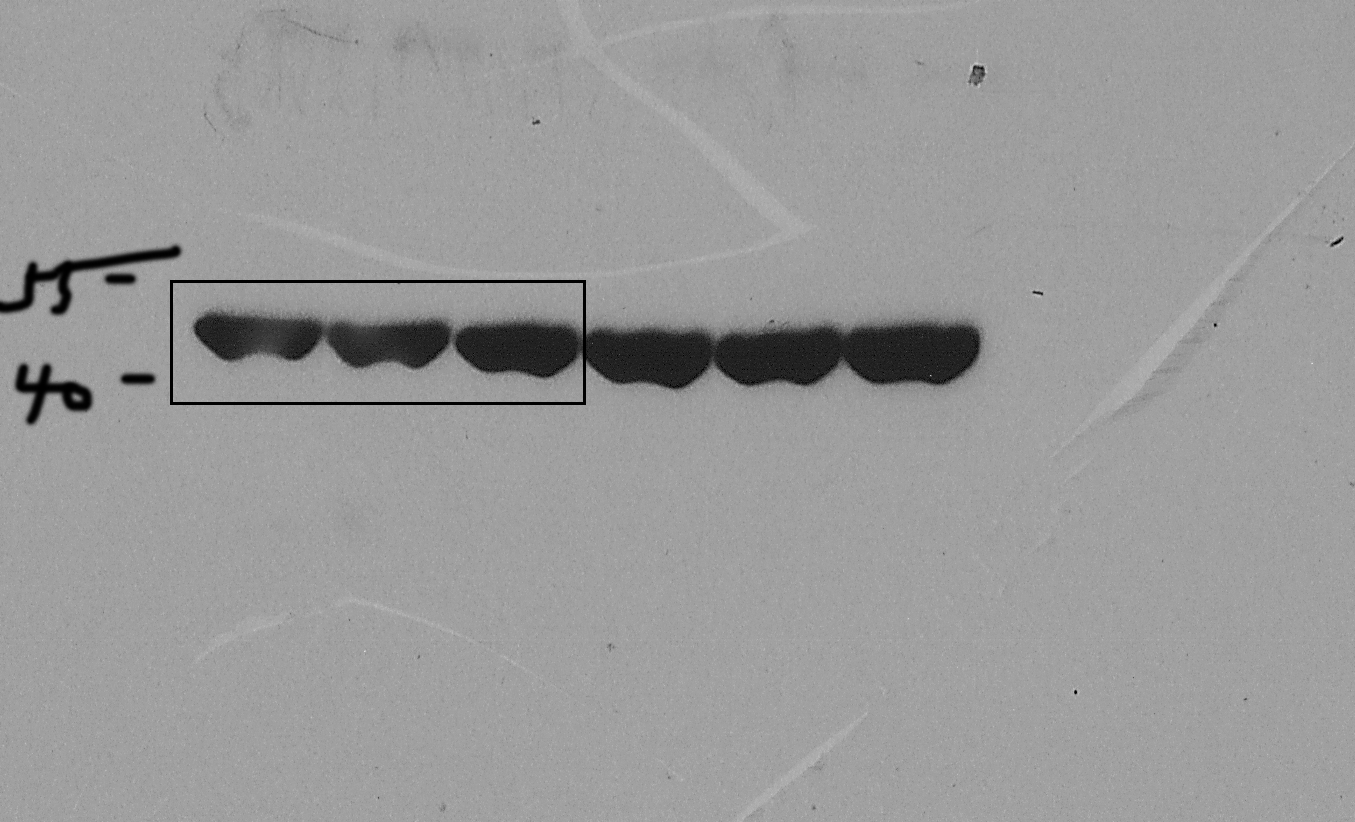

Supplement: Supplementary file 3 [file DataSheet2.ZIP › Fig 5/A/actin.TIF]

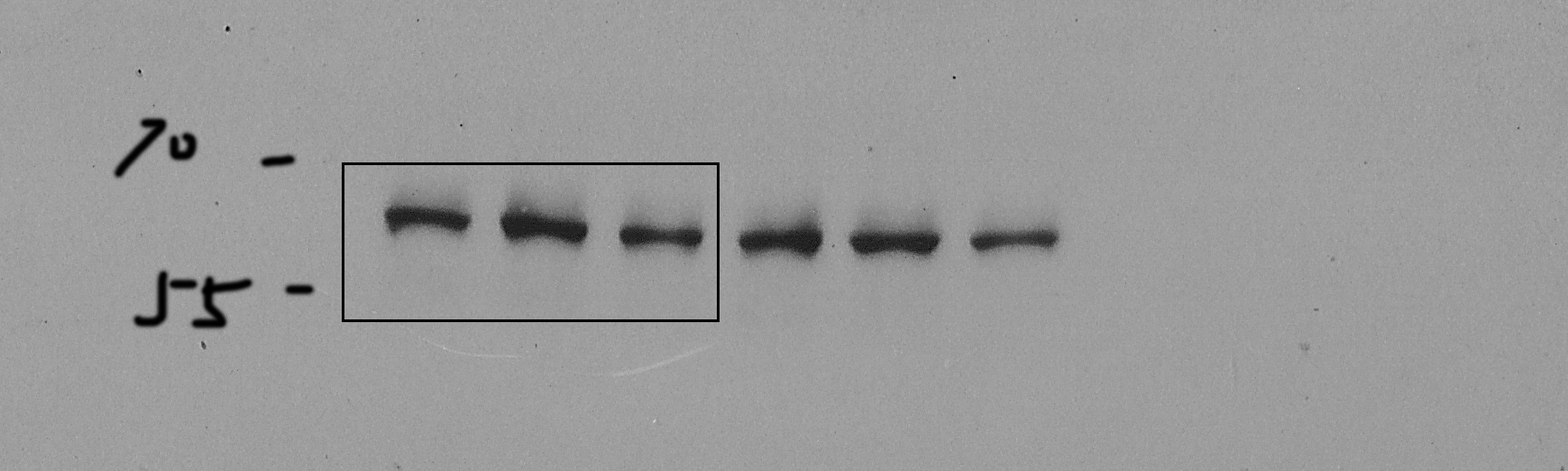

Supplement: Supplementary file 3 [file DataSheet2.ZIP › Fig 5/A/ampk.TIF]

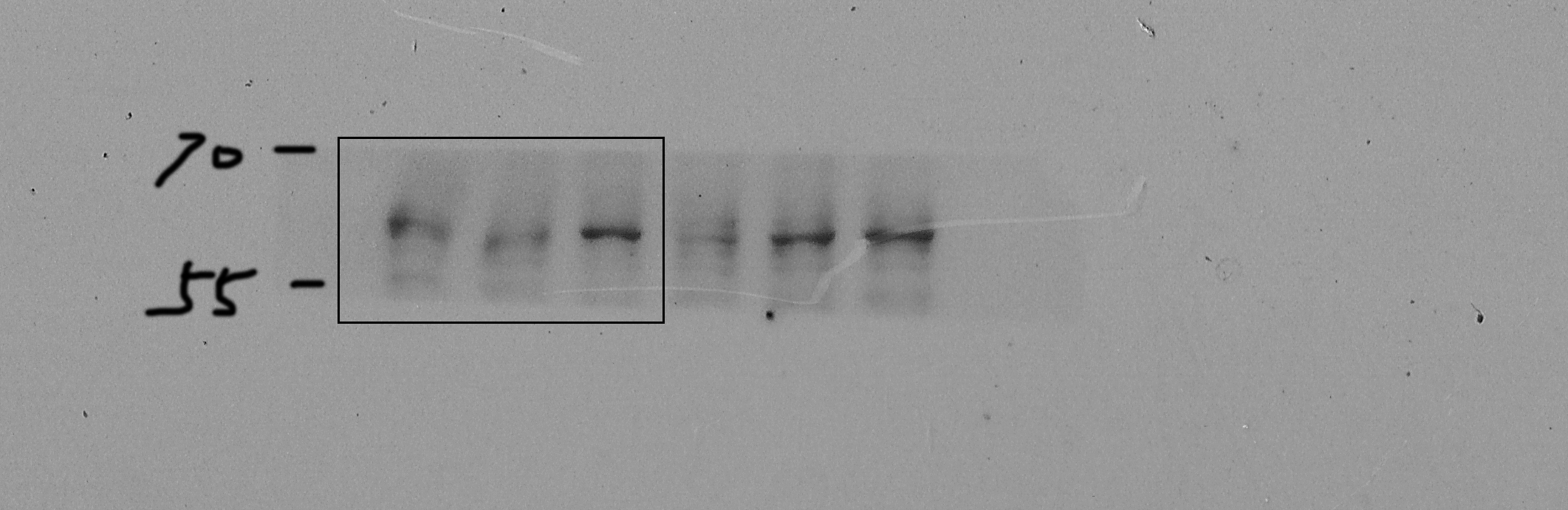

Supplement: Supplementary file 3 [file DataSheet2.ZIP › Fig 5/A/p-ampk.TIF]

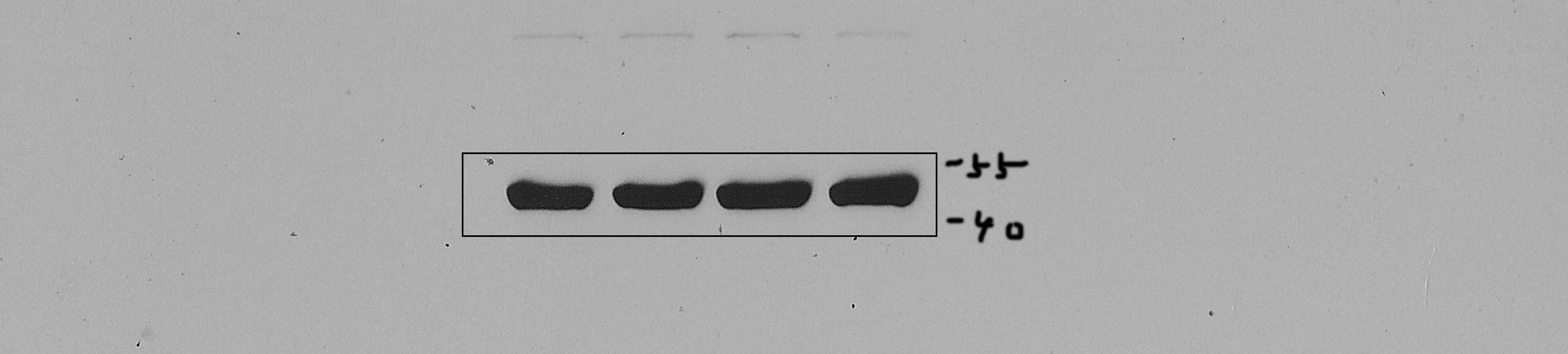

Supplement: Supplementary file 3 [file DataSheet2.ZIP › Fig 5/B/actin.TIF]

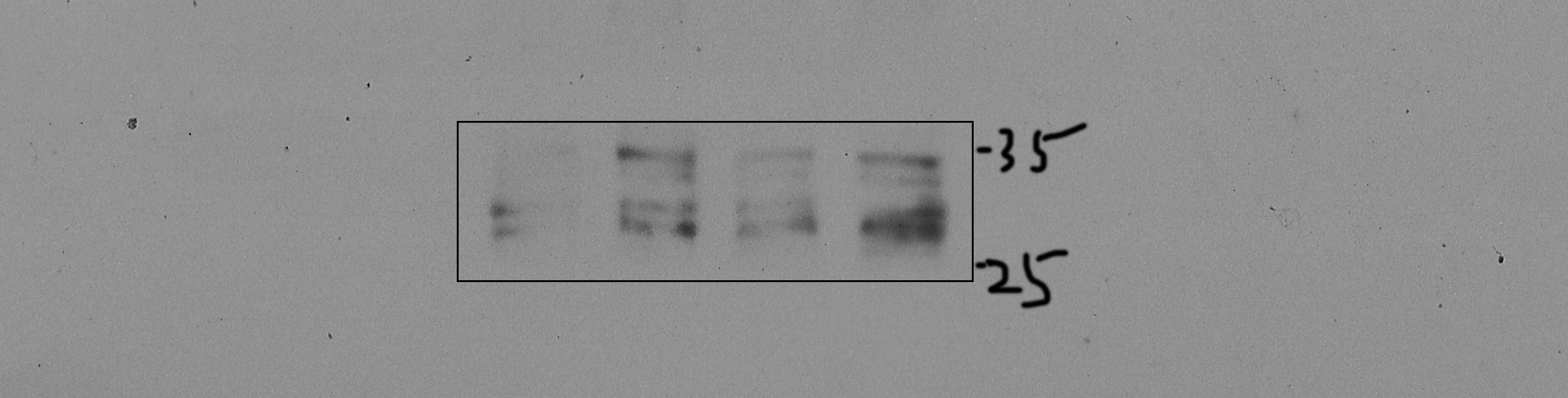

Supplement: Supplementary file 3 [file DataSheet2.ZIP › Fig 5/B/il1beta.TIF]

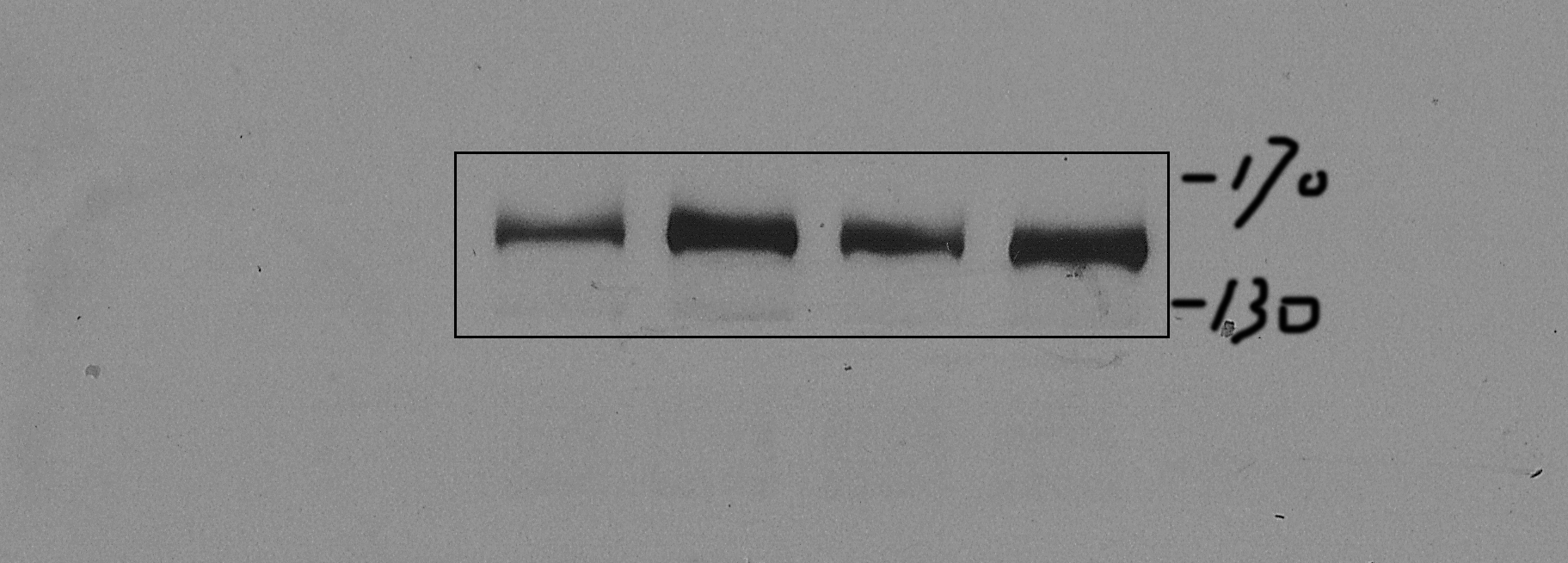

Supplement: Supplementary file 3 [file DataSheet2.ZIP › Fig 5/B/p-ulk.TIF]

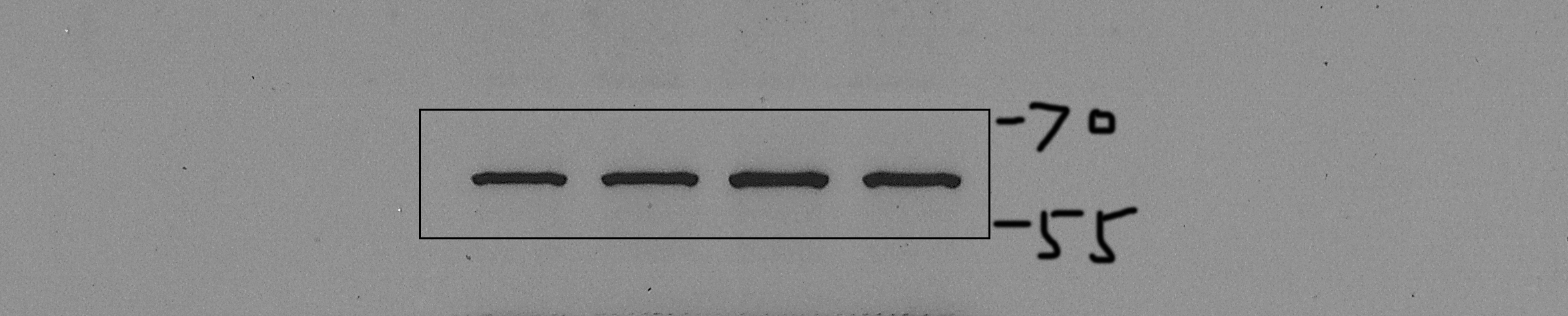

Supplement: Supplementary file 3 [file DataSheet2.ZIP › Fig 5/B/p65.TIF]

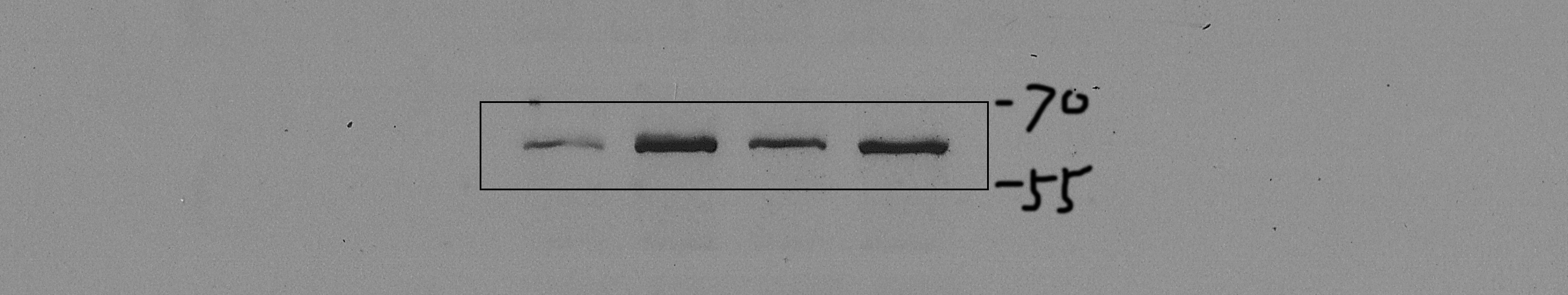

Supplement: Supplementary file 3 [file DataSheet2.ZIP › Fig 5/B/pp65.TIF]

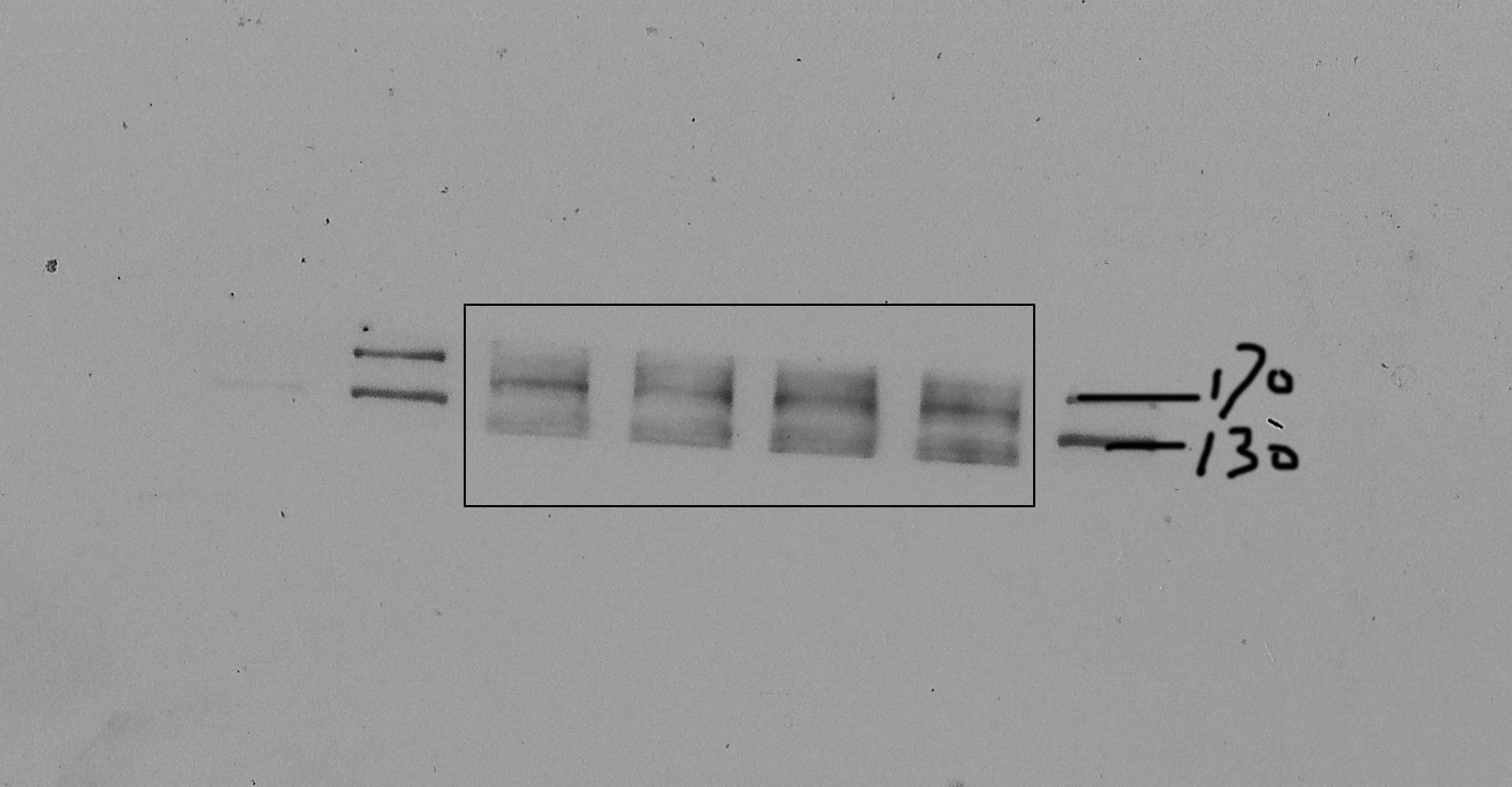

Supplement: Supplementary file 3 [file DataSheet2.ZIP › Fig 5/B/ulk.TIF]

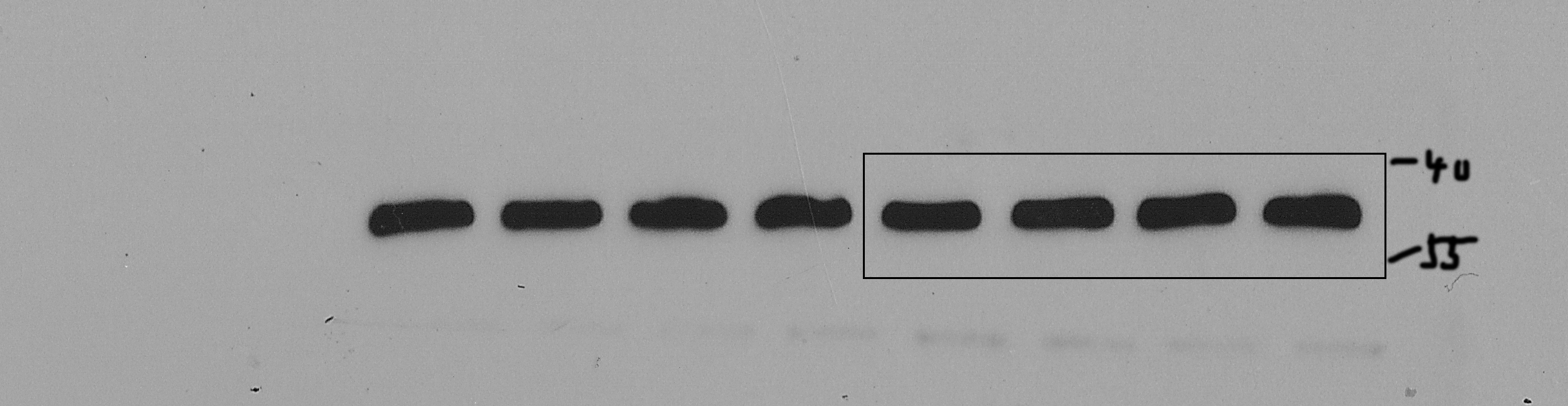

Supplement: Supplementary file 3 [file DataSheet2.ZIP › Fig 4/A/actin.TIF]

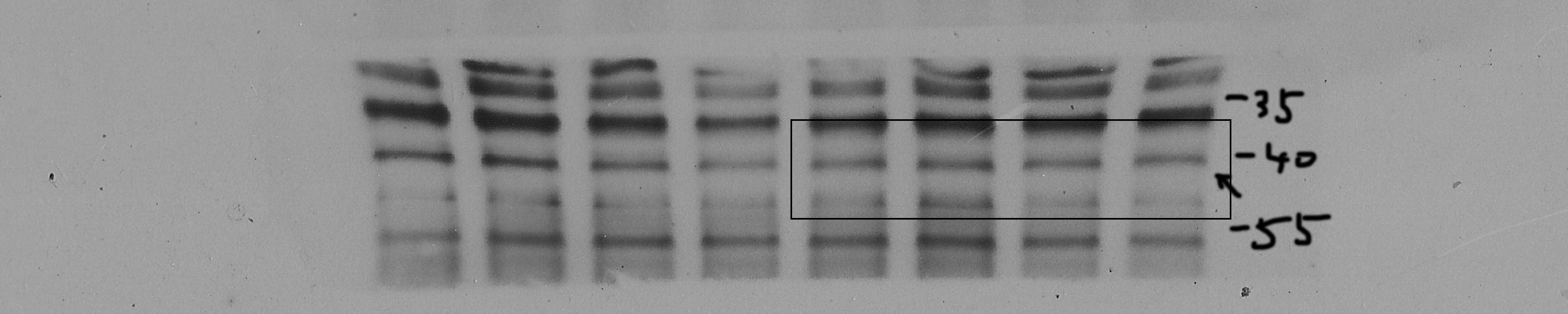

Supplement: Supplementary file 3 [file DataSheet2.ZIP › Fig 4/A/Bif-1.TIF]

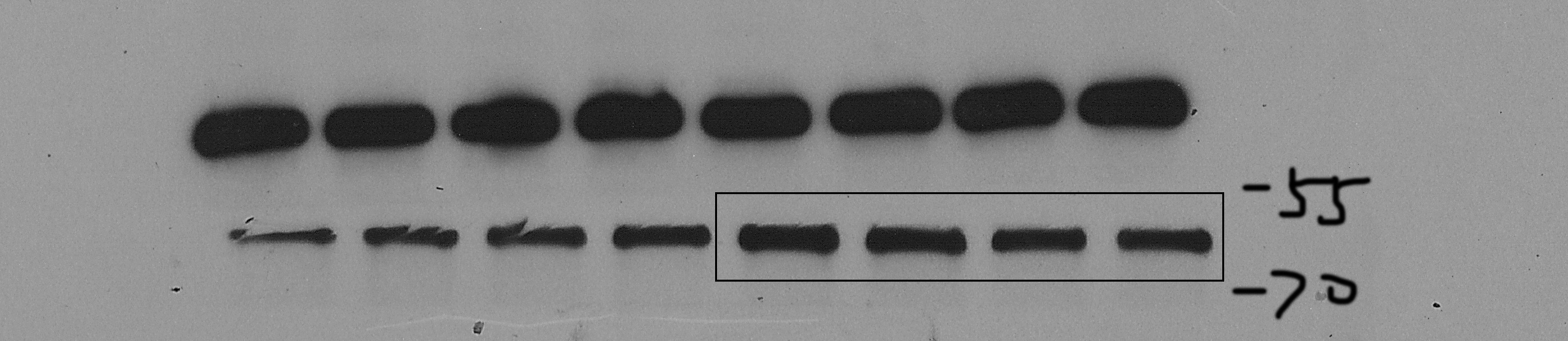

Supplement: Supplementary file 3 [file DataSheet2.ZIP › Fig 4/A/p62.TIF]

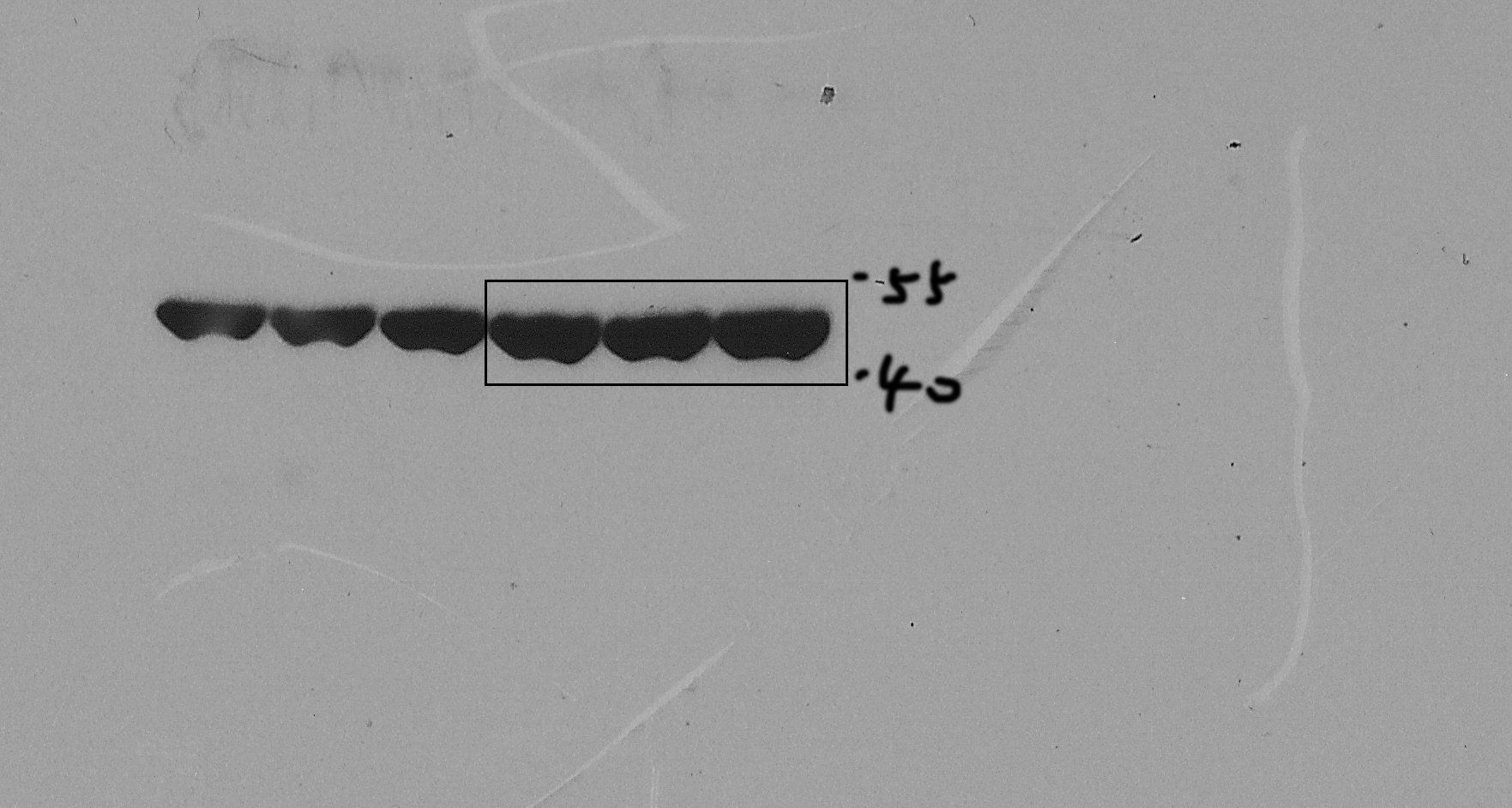

Supplement: Supplementary file 3 [file DataSheet2.ZIP › Fig 4/B/actin.TIF]

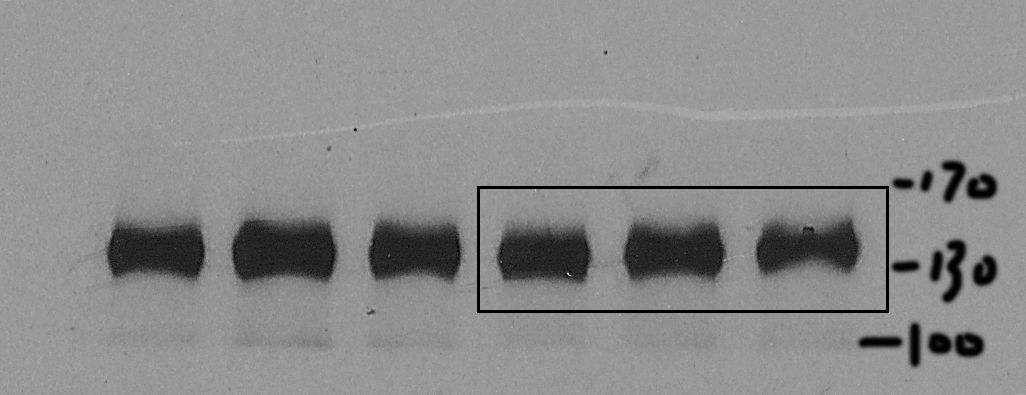

Supplement: Supplementary file 3 [file DataSheet2.ZIP › Fig 4/B/p-ulk.TIF]

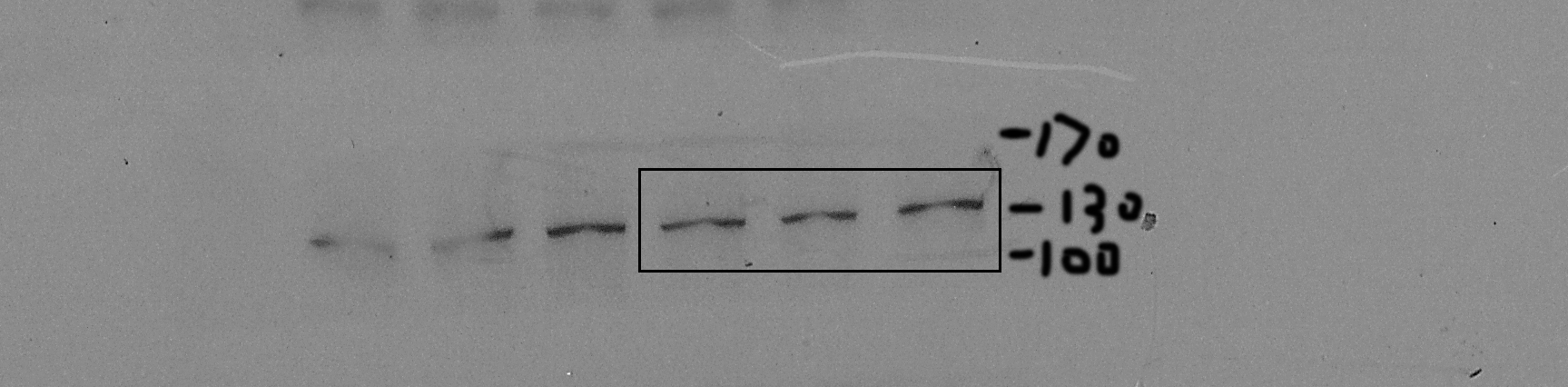

Supplement: Supplementary file 3 [file DataSheet2.ZIP › Fig 4/B/ulk.TIF]

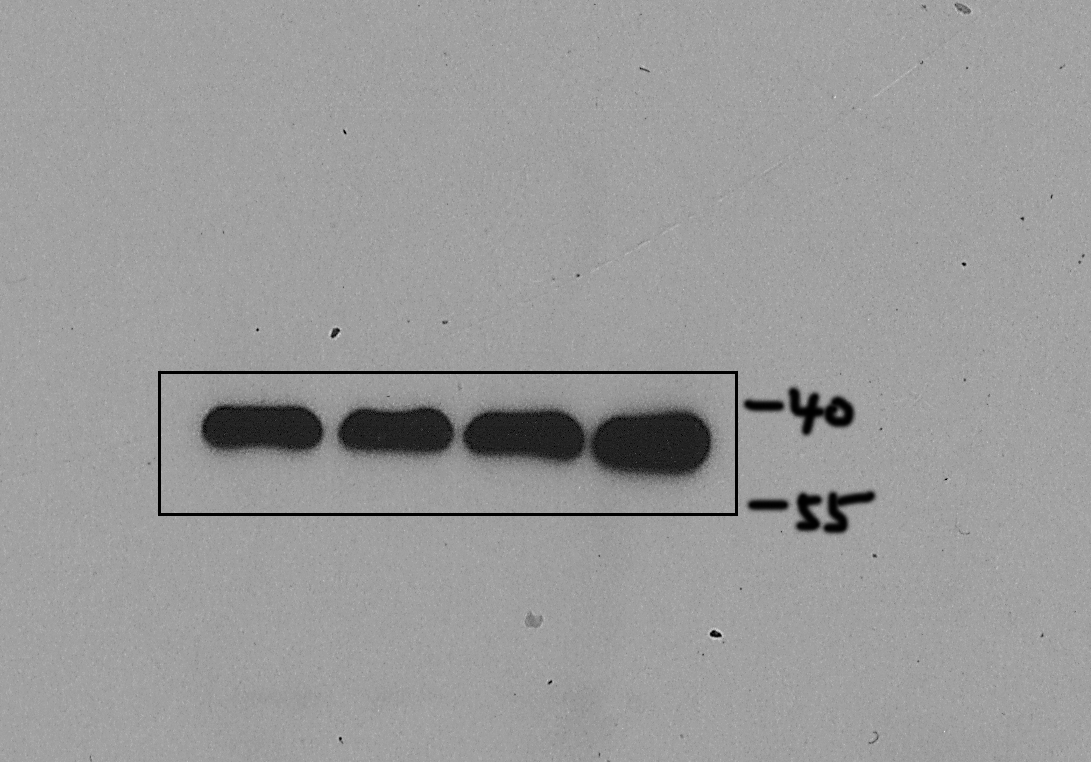

Supplement: Supplementary file 3 [file DataSheet2.ZIP › Fig 4/C/actin.TIF]

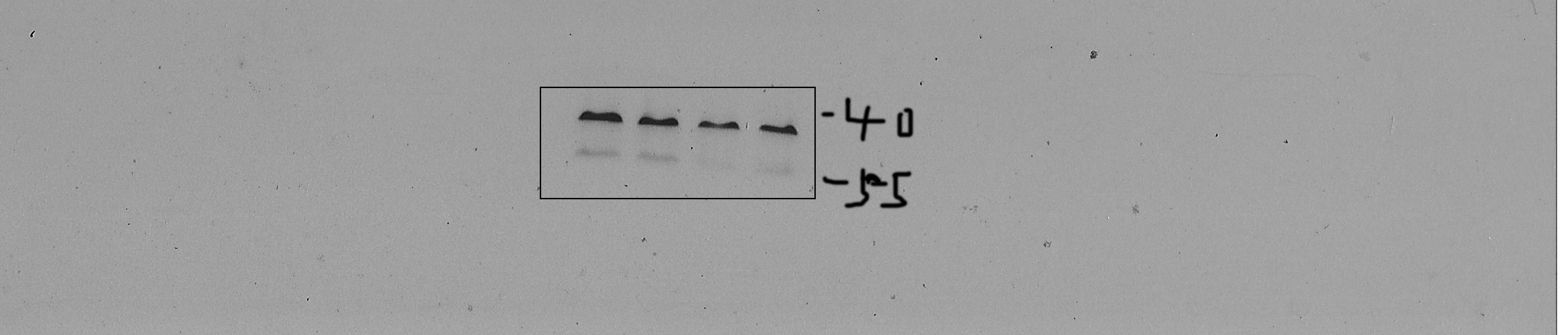

Supplement: Supplementary file 3 [file DataSheet2.ZIP › Fig 4/C/Bif-1.TIF]

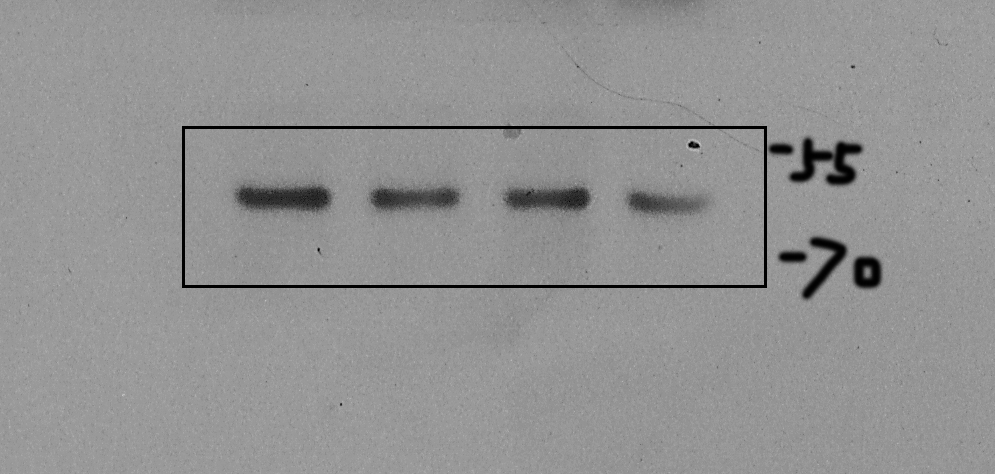

Supplement: Supplementary file 3 [file DataSheet2.ZIP › Fig 4/C/p62.TIF]

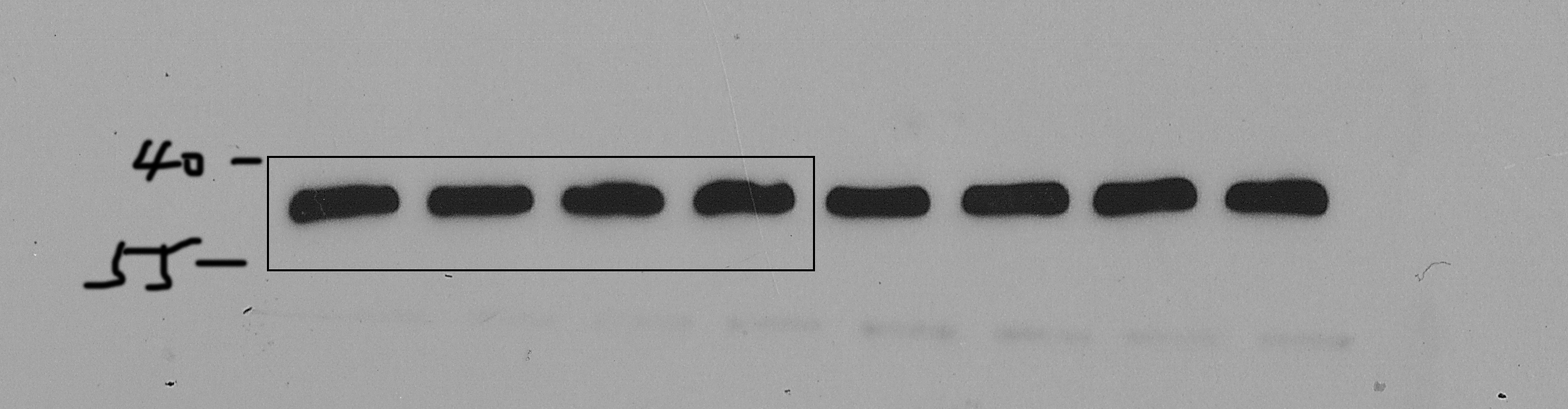

Supplement: Supplementary file 3 [file DataSheet2.ZIP › Fig 4/D/actin.TIF]

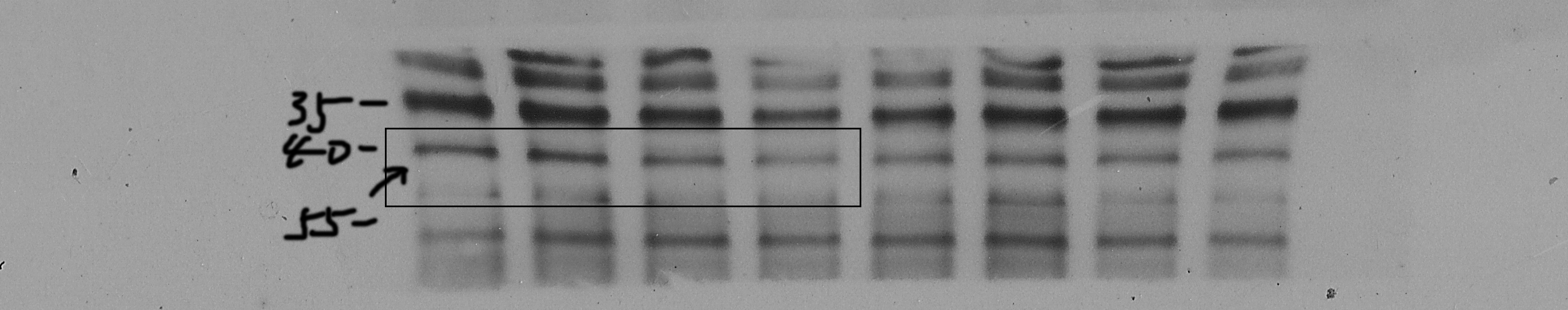

Supplement: Supplementary file 3 [file DataSheet2.ZIP › Fig 4/D/Bif-1.TIF]

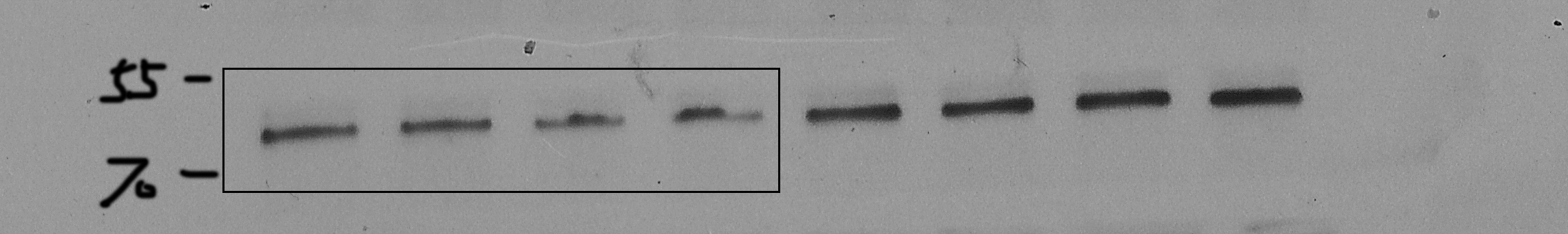

Supplement: Supplementary file 3 [file DataSheet2.ZIP › Fig 4/D/p62.TIF]
